# Mechanical or thermal damage: differentiating between underlying mechanisms as a cause of bone fractures

**DOI:** 10.1007/s00414-022-02825-x

**Published:** 2022-04-30

**Authors:** Divya S, Tristan Krap, Wilma Duijst, Maurice C. G. Aalders, Roelof-Jan Oostra

**Affiliations:** 1 Department of Biomedical Engineering and Physics, Amsterdam UMC, Location AMC, Amsterdam, The Netherlands; 2 Faculty of Law and Criminology, Section Forensic Medicine, Maastricht University, Bouillonstraat 3, 6211 LH Maastricht, The Netherlands; 3 Department of Medical Biology, Section Clinical Anatomy and Embryology, Amsterdam UMC, Location AMC, Amsterdam, The Netherlands; 4 Ars Cogniscendi Foundation for Legal and Forensic Medicine, Wezep, The Netherlands

**Keywords:** Bone, Heat, Blunt force trauma, Fracture, Forensic anthropology

## Abstract

To investigate the differences between pre- and post-fire fractures, 30 human forearm bones were subjected to either blunt-force impact, burning, or both. Bones, covered in soft tissue and wrapped in clothing, were burned in a reconstructed house fire. The burning context and dynamics led to differential burning, that was equal amongst the three groups. To evaluate the damage to the bones, a data collection sheet was developed based on the current literature on fracture features. To analyze the relation between exposure temperature and fracture class and occurrence, color was measured to estimate the exposure temperature. Observable and measurable changes on bone, and fracture surfaces, were macro- and microscopically analyzed. Many features overlapped between the three groups, and thus are not usable for differentiation. Fractures caused by blunt force impact (post-mortem, pre-fire) showed a rough fracture surface with smoothness in curved/slanted regions near the margin after burning, while heat-induced bone fractures showed a smooth fracture surface. The margins and surface of bone fractures that originated after the fire (indirect heat-induced) were evenly discolored, whereas heat-induced bone fractures showed uneven discoloration of the fracture margin and surface. Although there were generally more heat-induced fractures in the 450–700 °C range, no other distinctive trend was observed between exposure temperature and class of fractures.

**Supplementary Information:**

The online version contains supplementary material available at 10.1007/s00414-022-02825-x.

## Introduction

The destructive effect of fire is often used to conceal offenses and related evidence. In forensic cases, involving burned human remains, it is critical to determine the presence of peri-mortem damage and reconstruct the pre-burning events. Remains recovered from a context such as a house or car fire can include different fracture classes, relating to the moment that the damage occurred. Four different classes can be identified:

- ante- or peri-mortem, traumatic, bone fractures,
- post-mortem (pre-fire), non-traumatic, bone fractures (nTBF),
- heat-induced bone fractures (HIBFs),
- post-mortem during fire or post-fire bone fractures (indirect heat-induced bone fractures, iHIBFs).

A major challenge in fracture interpretation from thermally affected bodies lies in differentiating between these classes of fractures [1, 2]. The difficulty in differentiating between the fracture classifications arises from characteristics of one fracture class mimicking or showing similarities with the other classes [1, 3, 4]. Ante- to peri-mortem, traumatic, bone fractures can only be distinguished from post-mortem (pre-fire) bone fractures based on markers of vitality and thermal degradation can hamper such an analysis greatly [3]. HIBFs are a direct result of heat-induced (HI-) changes of the human body that is being burned including HI-changes within the bone matrix leading to loss of integrity. After thermal exposure, the bone can be subjected to forces or influences, which can lead to post-fire fractures. These fractures are not directly caused by exposure to heat but indirectly the result of structural and molecular HI-changes of the bone matrix, in combination with forces or influences acting on the bone [1, 5]. In some studies, these fractures are classified as situational. These fractures are termed iHIBFs in this study. Careful post-fire handling of the remains is key; incorrect handling is one of the possible causes of iHIBFs. Lastly, iHIBFs can co-exist with fractures from the other classes, either as independent fracture paths or merged with each other [6] (see Table S1 in Electronic Supplement, ESM, Section 1 for these features and studies that investigated them).

Blunt force trauma is one of the most common types of trauma amongst cases of death involving trauma, worldwide [7–9]. Blunt force trauma is typically caused by slow-loading forces impacting on a relatively large surface area and causes multiple types of fractures like complete simple, comminuted, or butterfly fractures [9, 10]. In contrast to blunt force trauma, HIBFs occur from disseminated, static stress within bone, or pulling forces from retracting soft tissue due to dehydration and carbonization [11]. Additionally, fire is dynamic, resulting in variation in temperature and exposure duration, and thus affects the material burning in different ways. Bones subjected to excessive heat will undergo several modifications like shrinkage, warping, superficial cracking (also referred to as *craquelle*), weight loss and deformation, fracturing, compositional, color, and dimensional changes [11]. Generally, bone goes through four stages when exposed to heat. Firstly, dehydration of the bone occurs first with hydroxyl bonds being broken and water removed from the bone. Secondly, pyrolysis of organic constituents (decomposition) occurs. Thereafter, representing the third stage, loss of carbonates and crystals conversion (inversion) causes calcination. The fourth stage involves fusion, the melting, and coalescing of inorganic crystals, causing dimensional changes [11, 12]. The different temperatures at these four stages cause changes in chemical composition of the organic constituents, leading to color changes of bone, namely from ivory white to brownish-black, to black then gray, and finally white [13]. Moreover, dehydration, collagen denaturation, and degradation reduce bone elasticity, resulting in deformation. As a result of deformation, HIBFs can occur in a variety of fracture types.

The strength of bone to resist external impact and the failure point leading to a fracture differ for every bone type and depend on the type of external force as well as the state of the bone [10]. Additionally, the necessary burning temperature, duration, and pattern, for a certain HI-change, differ for various bone types [3]. Currently, there are a limited number of studies available that discuss the effect of fire on bones with ante- or peri-mortem bone fractures using sufficient sample size [14–17]. Only a few studies are dedicated solely to distinguishing blunt force impact (BFI) fractures from HIBFs [1, 8, 18] (see Table S2 in ESM Section 1 for an overview of the major features of post-mortem (pre-fire) bone fractures corresponding to ante- or peri-mortem fractures and HI-fractures observed in former studies). For most of these latter studies, defleshed non-human skeletal material was used. Although homologous to human bones, the mineral density, hardness, and microstructure of non-human bones ultimately differ from those of human bones [3]. Hence, fracture propagation differs between human and non-human bones, making translation of results from non-human studies for actual casework involving human bodies unreliable [10]. Moreover, prior studies explored features, as listed in Table S2 in ESM Section 1, either as an individual feature or in small groups of features. The individually studied features concern color changes, microscopic morphological changes, or dimensional changes while the groups of features include fragmentation, fracture surface morphology (the exposed surface of the fracture), outline (the shape of the fracture lines, i.e., transverse or diagonal), and angle of fracture [8, 11, 13]. For these studies (but not limited to), unstandardized and/or ambiguous descriptions for features such as “slightly transverse” or “roughly V-shaped” or “right angle” were used [8]. Clear, consistent, and standardized descriptions would increase the objectivity of fracture feature analyses. Such analyses could then meet the Fry and Daubert standards for admissibility in court, more readily [19–21].

The goals of this study were to identify usable features to differentiate between post-mortem (pre-fire) bone fractures, HIBFs, and iHIBFs, determine the prevalence of these features, and assess if these features overlap. Data collection sheets were developed for this study (Fig. S3a and S3b in ESM Section 1). The effectiveness of these sheets for fracture analysis, and differentiation between the previously mentioned classes, was evaluated. To achieve the set goals, a burning experiment was carried out, mimicking a common house fire, in which human forearms, with and without post-mortem fractures caused by BFI, were burned. The sample collection consisted out of which, two of in total 19 human forearms divided over three groups, of which two were exposed to fire and one served as a control only containing fractures caused by BFI. The resulting fractures were examined both macro- and microscopically. The findings of the current study were compared with previous studies to assess the degree of agreement on the identified features.

## Materials and methods

For the meaning of abbreviations, see the list at the start of this manuscript, and for explanation of the used terminology, see the glossary in ESM Section 11, Table S26.

### Sample material and sample processing

Thirty-eight fresh-frozen human cadaveric radii and ulnae (19 radii and 19 ulnae) were collected from the body donation program of the Department of Medical Biology of the Amsterdam University Medical Centre, location Academic Medical Centre (AMC), The Netherlands. The bodies, from which the specimens were taken, were donated to science in accordance with Dutch legislation and the regulations of the medical ethical committee of the AMC (see the section compliance with legal and ethical standards at the end of this manuscript). The forearms were collected and stored at − 20 °C prior to the study, thawed, and subsequently manually defleshed with a scalpel and stored at 4–8 °C throughout the course of the study. Population data (age, sex, and bone dimensions) were collected; 22 bones originated from females and 16 bones from males; for further details, see Table S4 in ESM Section 2. Since age and medical history of the cadavers are confounding factors [8], the selection of tissues was based on the following criteria; all donations fell in the “old” adult category (50 + years, based on Buikstra and Ubelaker, 1994, [22]), without evident signs of osteoporosis (i.e., notable brittleness or strongly deviating weight) or known, from the medical history of the deceased, bone cancer, local surgery, known pre-existing or healed trauma, or other bone defects. The age ranged from 64 to 88. Eight out of the 38 bones were used in pilot studies (least weighing, to decrease variance in weight for the actual experiment) targeted at determining the optimal conditions and factors for (i) generating the BFI-fractures and (ii) burning of the bones—more details on the pilot study and results can be found in ESM Section 3. The conditions for the main experiment were set based on the results of the pilot study (ESM Section 3). The remaining 30 bones were used for the main experiment. Nitrile gloves were worn at all times during the study to prevent contamination.

### Grouping of bones

The 30 bones for the main experiment were split into 3 groups (Table 1) with 10 bones per group (see Table S8 in ESM Section 4 for specimens of each group). Sex, type of bone (radius/ulna), and dimensions were distributed as evenly as possible across the groups. In each group, 6 bones belonged to 6 different individuals whereas the remaining 4 bones (2 sets of forearms) belonged to 2 different donors (1 set from each cadaver) resulting in donations of 8 individuals to each group. The distribution of bones in every group is detailed in Fig. S9 and Table S10 in ESM Section 4.

**Table 1:** Grouping of forearm bones, including experimental grouping with corresponding sample size, each group with equal number of ulnae and radii

Group name (Code)	Type of exposure	Expected fracture classes	*N*
Control-burn (A)	Only burning & no BFI	HIBFs & iHIBFs	10
Control-BFI (B)	Only BFI & no burning	Post-mortem fractures (nTBFs)	10
BFI & burn (C)	Both BFI and burning	Post-mortem (pre-fire) fractures, HIBFs, and iHIBFs	10

### Blunt force impact production

**Fig. 1:**
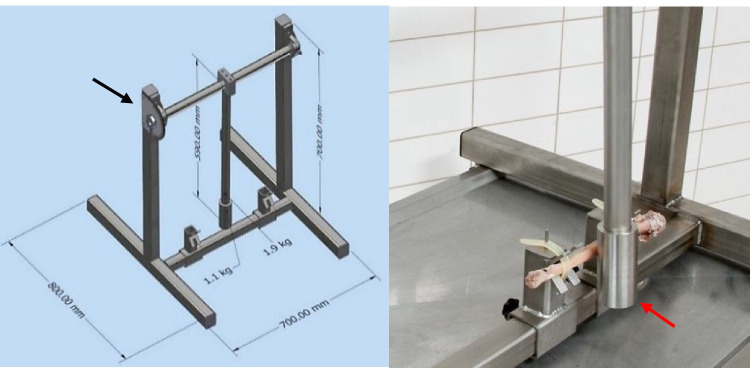
Custom-made pendulum-like contraption to produce fractures. The rod is attached to a cylindrical impactor (red arrow). A grading element (black arrow), ranging from (0° to 180°), shows the angle at which the rod was released from. Left: Schematic diagram of contraption with dimensions denoted. Right: Actual contraption with ulna bone in holder

Of the 30 thawed and defleshed bones, 20 bones (radii/ulnae) were fractured using a custom-made contraption resembling a pendulum apparatus (Fig. 1). The rod attached to a cylindrical impactor, which together weighted 3 kg and was 50 cm in length, mimicked the common weapon type involved in blunt force trauma cases such as a baseball bat [8]. The ideal angle and positioning of bone used on the contraption, for desired fracture production, were derived from the pilot study (see ESM Section 3). The rod was brought up to 80° and released at that angle, thereby creating a potential impact force of 2066 to 4650 N as a result from the kinetic energy (see ESM Section 3 (i) for more details).

Bones were positioned in pronation on the contraption, in a constant manner to standardize fracture production—posterior side of bones facing the impact rod with the radial/ulnar tuberosity facing away from the rod, middle of diaphysis centered, and head of radius/olecranon of ulna placed on the right holder (when facing the contraption from the front) (see Fig. 1). After BFI, each bone was reconstructed carefully for photographic documentation, using a DSLR Nikon D700 with a 35-mm F2.0 AF-D lens, and analysis.

### The burning experiment

**Fig. 2:**
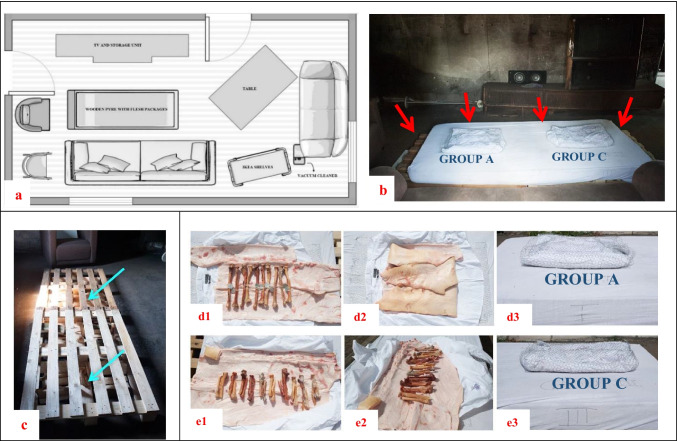
Burning experiment set-up. **a** Schematic representation of the room in firehouse that was used for burning the bones, including furniture and placement of wooden pyre. **b** Bone packages stacked on either side of mattress—group control-burn (*A*) on the left and group BFI and burn (*C*) on the right; packages and mattress on wood-wood pallet set-up; red arrows indicate positions of wooden blocks for fire ignition. **c** Mix of hardwood and softwood, depicted by cyan arrows, placed below wooden pallet within the firehouse; **d** and **e** Packaging process of bones—shirt unbuttoned and flattened on top of wire meshes, pig skin evenly spread on shirt, bones arranged on pig skin (**d1**, **e1**, **e2**), pig skin wrapped above bones (**d2**), shirt closed over pig skin, and buttoned up before wire meshes were tightened and tied together (**d3**, **e3**)

The bones were burnt at the Fire Station Soest, in a room of an isolated concrete firehouse, which resembled a basic residential living room, mimicking a context frequently encountered in forensic casework (Fig. 2). The burning experiment was conducted on a sunny day with mild wind, an average ambient temperature of 26.1 °C, and a relative ambient humidity of 65% at the time of the burning experiment. The pyre consisted of a mixture of hardwood (± 8 kg)-softwood (± 3.5 kg) under two (120 × 80 cm) wood pallets. A foam (polyurethane) mattress covered with cotton mattress-cover (70% cotton) was placed on the pallet. The two groups of bones, control-burn (A) and BFI and burn (C), were encased into “packages” using mesh-wire (hexagonal-shaped, 13 mm perimeter), 100%-cotton shirts, and pig skin (*Sus scrofa dom*., 15 kg and approximately 1.33 cm of adipose tissue as substitute for human skin with subcutaneous adipose tissue which serves as a fuel). In each package, all bones were covered together, as a group, by the skin and cotton. The entire set-up (Fig. 2a–e) was ignited using 4 cardboard paraffin-blocks of approximately 4cm^3^ each and a lighter. The windows and doors of the firehouse were opened for ventilation. The burning duration and temperature were derived from the pilot study (ESM Section 3). The bones were burned for approximately 50 min. The approximated temperature of the fire ranged between 700 and 800 °C, based on the potential temperature of the used fuels (mainly wood, the mattress, and pig skin). The actual temperature range was estimated during the experiment based on measurements by means of an infrared thermometer (PCE-890U, measuring range − 50 to 1150 °C, accuracy when measuring above 500 °C was ± 2 °C), and flame color. The fire was extinguished by a professional firefighter with a soft to mild water spray (3 min of spraying). The bones were carefully collected from the debris after a cooling down time of approximately 1 h, photographed positioned as the bones were found in situ, with a Nikon D700 with a 35-mm F2.0 AF-D lens, documented, and placed in plastic buckets for transportation to the institute (location AMC). Nitrile gloves were worn during salvaging. The samples were handled with tweezers, for safety of researchers and to prevent contamination of discoloration after the burning experiment. The residual water from the bones was removed by carefully blotting the bones with tissue paper. The bones were left to further cool and dry for 24 h before subsequent analysis in the laboratory.

### Data collection via macroscopic and microscopic analyses

**Fig. 3:**
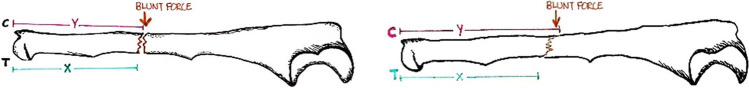
Schematic representation of measurement of fracture lengths. The fracture length is the length of the fracture line along the longitudinal axis of the bone. *Left*: fracture with transverse fracture outline; *right*: fracture with diagonal fracture outline. In both figures, X (blue line) depicts length of fracture on tension side (denoted by T) and Y (purple line) depicts length of fracture on compression side (denoted by C). Ratio of length of fracture on tension side to compression side is calculated by dividing value of X by value of Y

All bones were again photographically documented after transportation from the firehouse to the institute and after each data collection step, to assess any damage to bone from handling throughout the fracture analysis process. Fractures (denoted as Fx in data) and their features (Table 2 and Fig. 3) were analyzed according to the data collection sheets derived from former studies (Fig. S3a and S3b in ESM Section 1). The descriptions of the features were made more specific to avoid ambiguity (see Glossary Table S26 in ESM Section 11). Macroscopic and microscopic observations of these bone fractures were made according to this data collection sheet.

**Table 2:** Different features examined after each phase of experiments—after BFI and after burning. Last column shows the features analyzed in both instances. Refer to Table S11 in ESM Section 5 (scoring) for the categories for each feature, or Table S28 in ESM Section 11 (glossary). *Fx*, fracture. *Refer to Fig. 3 for description of this feature. **Some studies refer to the margin as the “edge,” for example, Behrensmeyer (1978) or Bohnert [32, 36]

Post-BFI features	Post-Burning features	Examined features	
		Observable	Measurable
• Length of fx* • Ratio of length of fx on tension side to compression side* • nTBF type	• State of burning • Colorimetry • Length of bone • HIBFs and iHIBFs • BFI-fracture (nTBFs) after fire (group BFI & burn (C) only)	• Fx type • Location of fx on bone • Color and shape of fx margin (boundary between fx surface and periosteal bone surface **) • Color of fx surface • Fracture surface morphology (smooth vs. rough) of fx surface • Number of fx on bone • Presence of fragmentation • Number of fragments • Fx category	• Fx angle • Size of fragments

The prevalence of features was evaluated, intra-group and inter-group, and the main comparisons are denoted in Table 3. Microscopic observations were made using the Olympus SXZ9 stereomicroscope, under × 8 to × 10 magnification. ImageJ software was used to analyze the features and determine angle of fractures, as described in the next sub-section.

**Table 3:** Intergroup comparisons and the different outcomes in relation to specifying features of each fracture

Compared groups	Outcome of comparison
Group control-burn (A) with group control-BFI (B)	Identification of specific features of HIBFs and nTBFs
Group control-burn (A) with group BFI & burn (C)	Determination of HIBFs and other overlapping features
Group control-BFI (B) with group BFI & burn (C)	Determination of BFI-fractures and other overlapping features
Post-BFI/ *pre*-fire group BFI & burn (C) with *post*-fire group BFI & burn (C)	Determination of similarities and differences of nTBFs and HIBFs features in the same bone

### Measurement of angles

**Fig. 4:**
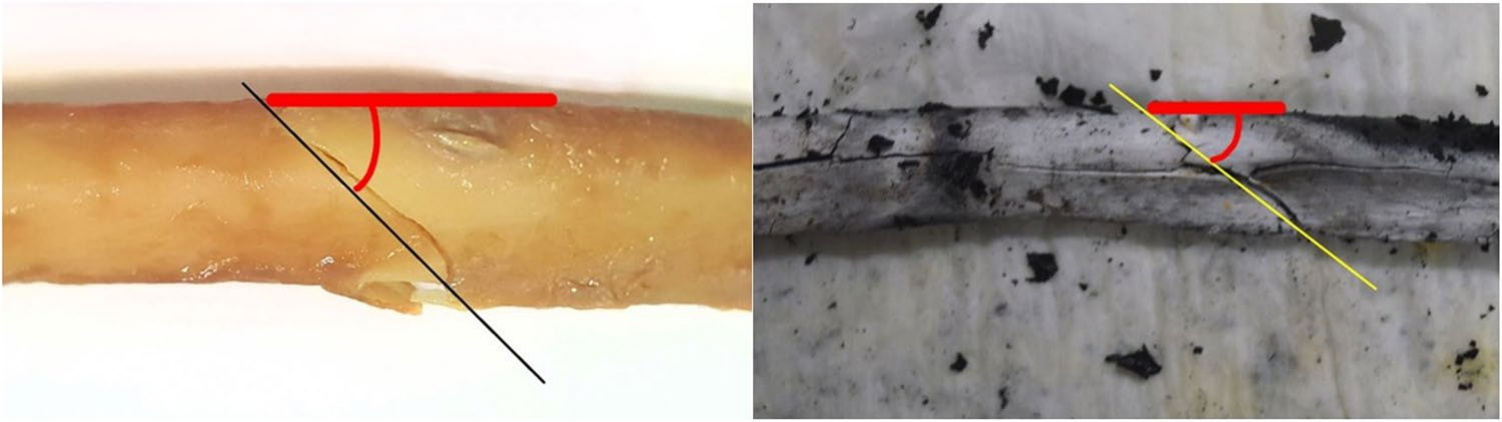
Angle measurement using ImageJ—negative slope (downhill) was drawn, black/yellow line, from compression to tension and angle, red line, determined. *Left*: bone subjected to BFI; *right*: burned bone. Images are of 2 different bones

For the angle of fracture, apart from microscopic observation, the photographs of the bones were used to draw a negative slope (downhill) across the proximal end of the fracture, spanning from the compression to tension side of the bone. Thereafter, the angle was measured as in Fig. 4, thrice (blindly) at different times, to account for observational errors. The mean angle for each bone was then determined. The standard error of measurement and the angle of fracture were calculated for each bone.

### Colorimetric analysis

**Fig. 5:**
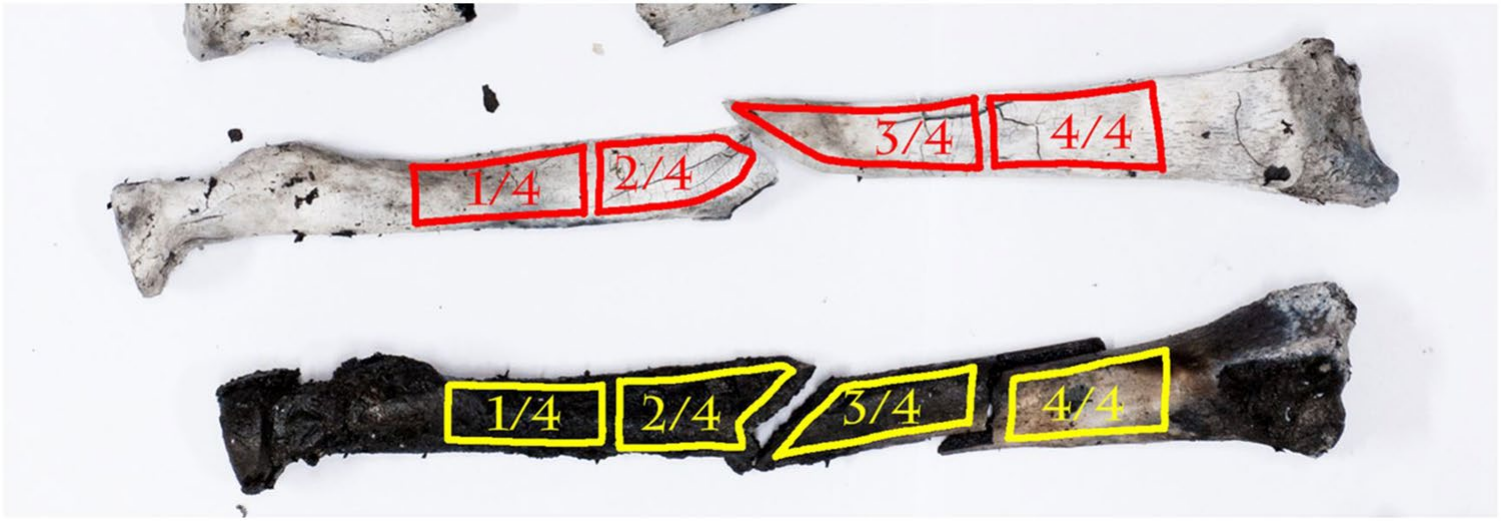
Sections used to measure L-b values − 1/4 proximal, 2/4 proximal (Fx proximal in BFI/nTBF bones), 3/4 distal (Fx distal in BFI/nTBF bones), and 4/4 distal. Each section is marked (red and yellow), slightly away from cortical bone margins. The values for 1/4 proximal and 4/4 distal were used to calculate the average temperature the bone was exposed to at the epiphyseal ends of the bone. The values for 2/4 proximal and 3/4 distal were used to calculate the average temperature the bone was exposed to at the intermediate region of the bone

The burned bones were photographed in the laboratory, 14 days after the burning experiment. Bones from groups control-burn (A) and BFI and burn (C) were arranged on 4 white sheets of paper and photographed from top view. Photographs were taken using a DSLR, Nikon D700 with Nikon 35 mm AF-D F2.0 and 24 mm AF-D F2.8 lenses, then adjusted for major white balance and converted to L*a*b color space (ImageJ – color transformer plugin) to determine the L (lightness) and B-coordinate. Lighting was equalized and the camera color calibrated with an X-rite ColorChecker Classic [13]. Due to brittleness of bones, the anterior portion of the bones was photographed. For each bone, 4 sections (Fig. 5) were manually selected to calculate the average L* and B* values for subsequent temperature estimation by means of the published model by Krap et al. (2019) [13]. The correlation between color changes and exposure temperatures from Krap et al. (2019) study was used to determine the phase of burning each bone was in (95% overall accuracy) [13]. The temperature ranges observed at each of the 4 regions were then used to calculate the average temperature of burning at the intermediate regions and epiphyseal (ends) regions of the bone (see Fig. 5). This is to account for possible temperature variations that might occur at these regions, caused by the differential exposure to heat during the burning. The fractures, at either region, were analyzed in terms of quantity and classification of fracture: post-mortem pre-fire fractures, HIBF, and/or iHIBF. This approach was aimed at identifying patterns occurring between exposure temperature and the types of fractures (see Table S11 in ESM Section 5) and their features at each region and across the bone.

### Statistical analysis and comparison with literature

Statistical analysis was performed in Microsoft® Excel and IBM SPSS Statistics 25. The performed group comparisons are shown in Table 3. Descriptive statistics was done for all groups.

Fracture features correspond to categorical (color, stage of burning, fracture outline, etc.) and numerical data (dimensional changes); therefore, Kruskal–Wallis test and one-way ANOVA analysis were done to test for significant statistical differences (*p* < 0.05) on features between groups. All the fractures in every bone, in all three groups, were analyzed and included in the statistical comparisons performed on the groups. These analyses enabled the identification of BFI/HI-specific features and/or any overlapping features. Due to the limited sample size of *n* = 10 per group and presence of repetitive values for some features, Q-Q plots were made to determine normality of data and a Levene’s test was done to test for homogeneity of variance, both being requirements for one-way ANOVA analysis. ANOVA was chosen over a *t*-test as it provides more information about the partitioning of variances and is more flexible with analyzing the data [23]. Kruskal–Wallis (KW) test was done to analyze the categorical data. The categorical data was numerically labeled/scored to aid in this analysis, as tabulated in Table S11 in ESM Section 5. Mann–Whitney (MW) test was performed to determine differences in changes in lengths of bones and ratios of lengths between unburned and burned group BFI and burn (C) bones. This test was also used to determine differences between number of fractures at the epiphyseal ends and those at the intermediate region. Kruskal–Wallis test was used for analyzing differences in fracture class (post-mortem pre-fire, HIBF, or iHIBF) at the epiphyseal and intermediate regions. Finally, the observed features were cross-compared with current literature, in terms of feature description and occurrence in each fracture class, to assess and explain similar and conflicting results.

## Results

### General observations for post-BFI fracture features—groups control-BFI (B) and BFI and burn (C)

After exposure to BFI, all 20 bones from group control-BFI (B) and group BFI and burn (C) (pre-burning state) showed at least one fracture and 15 out of these 20 bones showed fractures at the intermediate part of the bone (see Fig. S13–S14 in ESM Section 6 and Tables S22–S23 in ESM Section 10). Twelve out of 20 bones showed fragmentation varying in size. Out of the 20 bones, there were 11 bones with complete-comminute fractures and 9 bones with complete-simple fractures. All fractured bones showed a smooth fracture surface morphology. Helical/curved was the most common fracture outline (9 out of 20 bones), followed by transverse (5 out of 20 bones), diagonal (4 out of 20 bones), columnar (1 out of 20 bones), and longitudinal-transverse (1 out of 20 bones). Twelve out of 20 bones were oblique fractures, followed by spiral, transverse, comminute, and one segmental fracture. Group B resulted in greater fracture lengths (predominantly 10–15 cm) on the proximal side than the distal side of the bone, and vice versa was observed for group C. The ratios of fracture length of tension to compression side were greater on the proximal side than on the distal side of the bones for group B while the ratios were the same for either sides of group C. As expected, these ratios were nearly the same for proximal and distal sides of the bones with transverse fractures (see bone sample UB7 and UB9 in Table S22 in ESM Section 10). Only one bone (see bone sample RC7 in Table S23 in ESM Section 10) showed a segmental fracture, and thus, this bone was not used for statistical analysis in order to avoid large deviations in fracture types.

### Observations from burning experiment

The fire reached high temperatures, with a maximum of 800 ± 20 °C, about 25 min after ignition, when it spread and grew. It was stable for about 15 min, showed mainly bright red flames, before dying down for the last 10 min with consumption of fuel at the outskirts of the pyre, while the center still had fuel.

### General observations for post-Burning fracture features—groups control-burn (A) and BFI and burn (C)

Group control-burn (A) bones were evaluated as a group, with new specimen/sample numbers, due to the cominglement of bones during the burning experiment (Fig. S12 in ESM Section 6 and Table S24 in ESM Section 10). Group BFI and burn (C) bones were individualized using the fracture margins, to original specimen numbers, thus being easily comparable to pre-burning results (Fig. S14 in ESM Section 6, and Tables S23 and S25 in ESM Section 10).

**Fig. 6:**
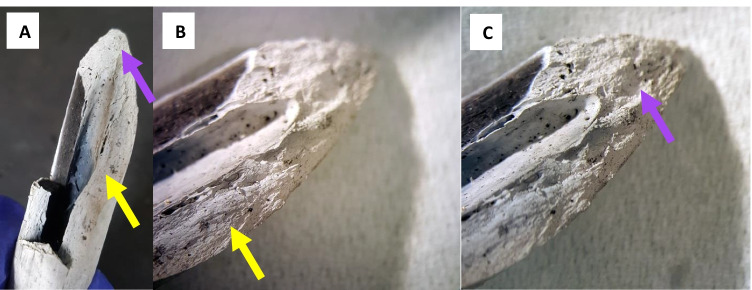
An oblique fx in the intermediate section, of a radius, in close perimeter to a stepped fracture on the opposite side (**A**). Both roughness and some smoothness (at sloped/curved margins) in burned bone from group BFI and burn were observed on the fx surface of the oblique fx. Yellow arrow indicates the relatively smoother region (**B**), and the purple arrow indicates the irregular, rough region of the fx surface near the margin (**C**)

Both groups showed fragmentation, but this feature was not statistically evaluated since some fragments were missing and some broken during post-fire recovery or further broken during transport to laboratory. Fifteen out of 20 bones showed large areas of partial calcination and small areas of carbonization; the remaining bones were predominantly carbonized with small regions of partial calcination. Four out of the remaining five bones were partly burnt and partly still relative fresh and the last one was carbonized. Moreover, the bone excluded for evaluation after post-BFI fracture analysis was not examined post-burning (see bone sample RC7 in Table S23 in ESM Section 10). The discoloration and state of burning of both groups did not show any distinct trend based on visual color assessment, although it seemed that calcination began from the intermediate portion of the bone and proximal ends were more carbonized than the rest of bone. Group A bones showed transverse fracture outlines (4 out of 5 bones which showed fractures post-burning), while group C bones mostly showed helical/curved (5 out of 20 bones) and diagonal fracture outlines (4 out of 20) comparable to the findings before burning (see previous section). Both groups exhibited fracture margins corresponding to margins observed after BFI; however, group A had smooth fracture surface whereas group C largely had rough fracture surface and some smoothness in curved/sloped regions of the fracture surface near the fracture margins (Fig. 6).

**Fig. 7:**
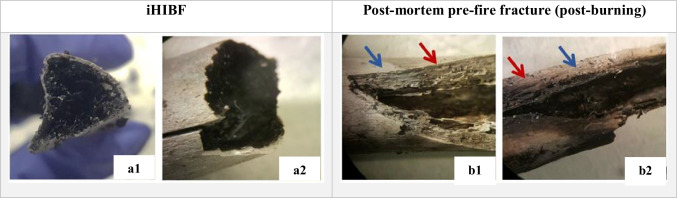
The difference in appearance and discoloration of fracture margins and surface for iHIBFs (**a1** and **a2**) and post-mortem pre-fire fractures (post-burning) (**b1** and **b2**). **a1** and **a2** show the contrast between white to gray discolored fx margins and carbonized fx surface and inner part. **b1** and **b2** show fracture surfaces with discoloration in a gradient from dark on the inside to lighter towards the periosteal surface, as represented by the blue and red arrows; blue indicating a lighter color and red indicating a darker color

Identified HIBFs were prominently longitudinal, followed by straight transverse and step HIBFs in both groups while iHIBFs were predominantly longitudinal. All bones were very brittle, with group C bones being more brittle than group A. There were less bones with iHIBFs in group A (6 out of 10) than group C (8 out of 10). iHIBFs showed more carbonization of the inner part of the bone and more calcination of the outer layers of the bone; this pattern was evenly spread across the fracture surface. The fracture margins of iHIBFs were mostly calcined, with an almost gradual pattern turning inwards from white to gray. The identified post-mortem (pre-fire) fractures showed less carbonization and more calcination of the inner layers of the fracture surface after burning. The fracture margins of the post-mortem pre-fire fractures displayed an uneven pattern of carbonization and calcination (see Fig. 7). The HIBFs were similar in discoloration pattern to the post-mortem, pre-fire fractures post-burning.

The fractures of both the HIBFs and iHIBFs penetrated into the medullary cavity, but one of the fracture margins of iHIBFs was elevated while those of HIBFs were in the same plane (Table 4). iHIBF near the BFI site showed these one-sided elevations as well. After reconstruction of diaphyseal sections, it became clear that longitudinal fractures with one-sided elevation appeared on both fragments of the post-mortem pre-fire fracture (post burning), with the elevated margin on the same side. HIBFs near the BFI site did not show any elevation.

**Table 4:**
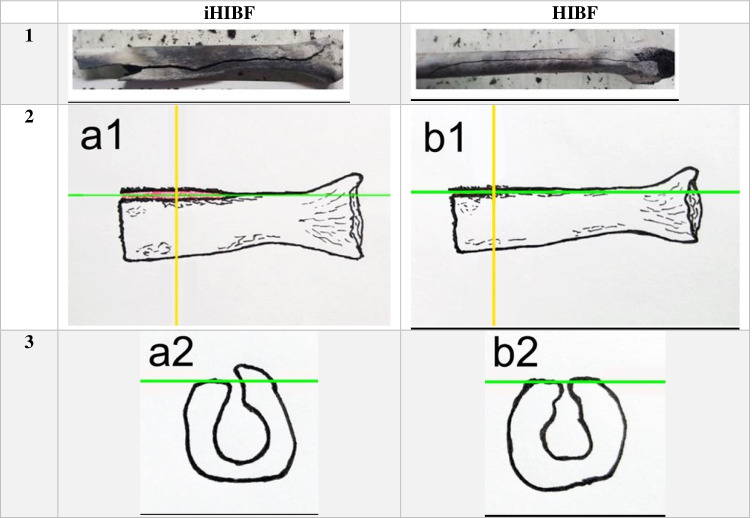
An elevated margin on one side of the iHIBFs and on corresponding level for HIBFs. 1st row overview, *left*: longitudinal iHIBF; and right: longitudinal HIBF. 2nd row: schematic eye-level representation of elevated margins in iHIBFs (a1) vs. margins on same level as longitudinal axis of bone in HIBFs (b1); 3rd row: schematic transverse view of margins of iHBIFs (a2) vs. HIBFs (b2). Green line indicates longitudinal plane and yellow line shows transverse plane

Heat borders were also observed close to the BFI sites of group C bones, denoting transition of burning phases of bone. Microstructural fractures were found in partially calcined areas of bones. Large, calcined bone fragments and corresponding smaller fragments could be pieced back together and reconstructed more easily than carbonized bone fragments with related smaller fragments. Since direct comparison of bones was not possible, the combined data of unburned bones was compared with the combined data of burned bones of group C. Most burned bones exhibited blunt fracture margins; thus, the sharp edges resulting from BFI had lost its sharpness. The burned group C bones mostly showed longitudinal and some stepped HIBFs. The iHIBFs observed in group C were largely longitudinal fractures with elevated fracture margins on one side. As for bone dimensions, most bones in group C exhibit shrinkage. Only one bone (UC10), however, showed increased lengths for both proximal and distal part of the bone, denoting an expansion across the bone (see Table S25 in ESM Section 10). The ratios of length on tension to length on compression for proximal and distal bone had mostly decreased.

### Colorimetric analysis

**Fig. 8:**
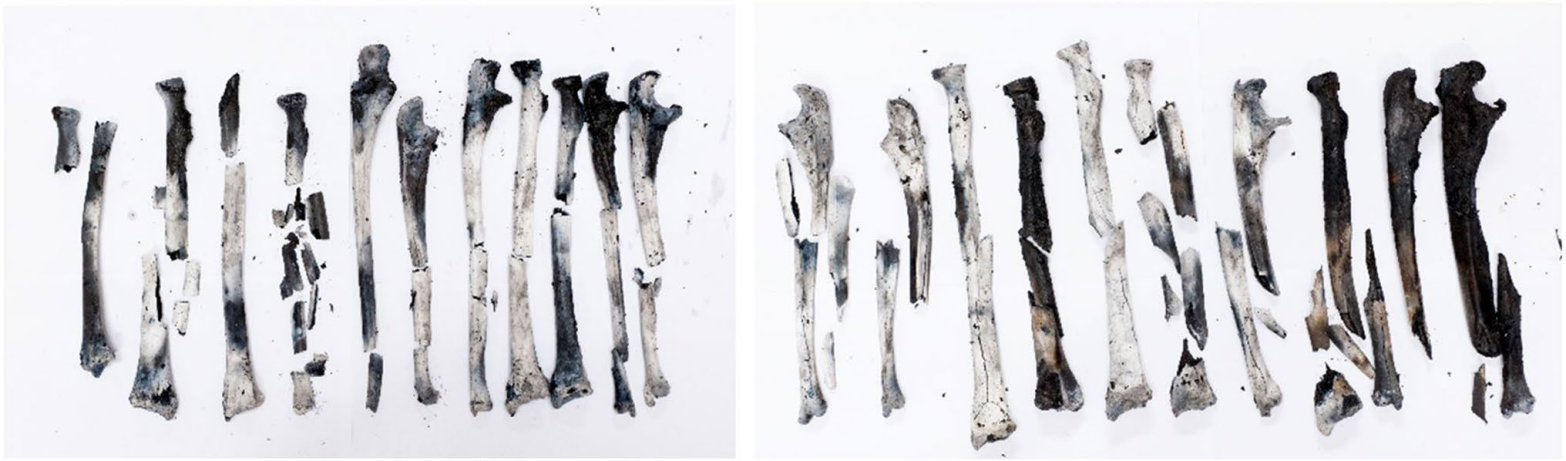
Group control-burn (**A**) on the left and group BFI and burn (**C**) on the right, taken after burning

**Fig. 9:**
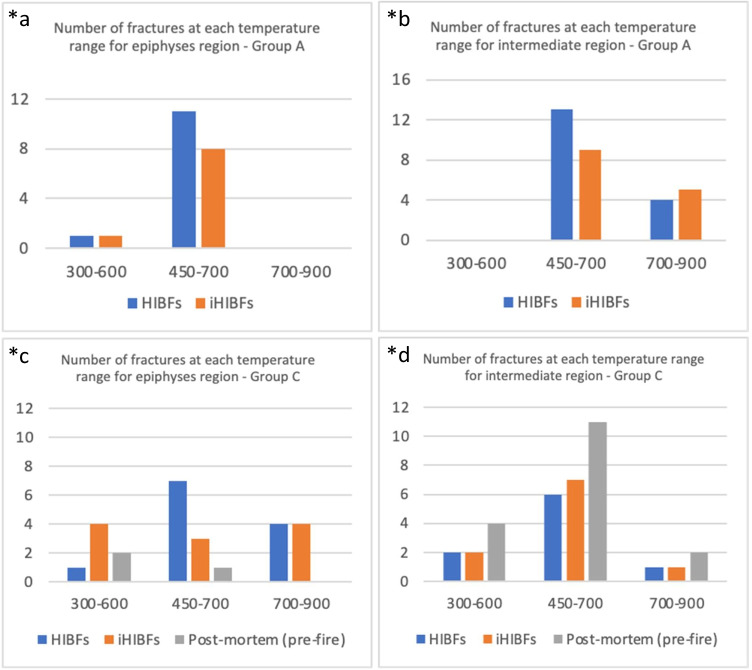
Bar graphs to illustrate the number of fractures found within each estimated temperature range at the epiphyses and intermediate regions of group control-burn (**A**) bones (*a, *b) and of group BFI and burn (**C**) bones (*c, *d). Left: shows number classified fx seen at the epiphyseal region (*a, *c). Right: shows number of classified fx seen at the intermediate region (*b, *d). Fracture class post-mortem (pre-fire) was assessed solely for group C

From visual observation, the majority of the bones showed both carbonization and calcination (Fig. 8). Based on the colorimetric model published by Krap et al. (2019), the majority of bones in group control-burn (A) and BFI and burn (C) were estimated to have been exposed to a maximum temperature of 450 to 700 °C (Tables S15–S16 in ESM Section 7); this shows the equality in burning conditions [13]. There were more fractures in the intermediate region than at the epiphyseal ends for both groups. HIBFs were the most common class of fractures across bones in group A (Table S17 in ESM Section 7). HIBFs and iHIBFs were the most common classes of fractures across bones in group C (Table S18 in ESM Section 7). In group C, there were more HIBFs and iHIBFs seen at the epiphyseal ends and more post-mortem (pre-fire) fractures at the intermediate region. There were more fractures in the intermediate region than at the epiphyseal ends for both groups control-burn (A) and group BFI and burn (C) (see Fig. 9a–d) (for further details, see Fig. S19–S20 in ESM Section 7). There seemed to be more carbonization, than calcination, at the epiphyses of most bones amongst both groups. No distinctive trend has been found when comparing the temperature of burning at the intermediate and epiphyseal ends and the classes of fractures (post-mortem pre-fire, HIBFs, or iHIBFs) observed at these regions.

### Statistical analyses of BFI and HIBF features between groups

Numerical data met the requirements for ANOVA, Q-Q plots exhibited normality, and Levene’s test showed homogeneity of variances. The categorical data failed to meet the ANOVA requirements; since the observations were independent of one another, the KW-test was performed. Table 5 shows the results from all statistical analyses which are discussed in detail below. Data on the dimensional changes can be found in the ESM Section 8.

**Table 5:** Results from ANOVA and Kruskal–Wallis analyses for the various comparisons between groups control-burn (A), control-BFI (B), and BFI and burn (C). The degree(s) of freedom for each analysis was 1. * indicates a statistically significant result (*p*-value < 0.05). *Fx* fracture

Groups compared	Fracture feature analyzed with ANOVA or MW	Sig	Fracture feature analyzed with KW test	Asymp. Sig
*Post-BFI:* Groups B vs. C (pre-burning)	Fx length, proximal bone	0.986	Fx surface	1.000
	Fx length, distal bone	0.983	Fx outline	0.206
	Ratio of length, proximal bone	0.872	Type of Fx	0.042 *
	Ratio of length, distal bone	0.842	Fx category	0.849
	Fx angle	0.007 *	Location on bone	0.039 *
			No. of fragments	0.719
			Fragment size	1.000
*Post-burning:* Groups A *vs.* C	Fx angle	0.658	Burning state	0.206
			Top color	0.450
			Second common color	0.529
			Third common color	0.735
			Fx surface	0.000 *
			Fx outline	0.038 *
			Fx category	0.003 *
			Location on bone	0.007 *
			Most common fire	0.051
			Second common fire	0.962
			Not directly fire	0.322
*Pre- vs. post-burning* *Group C*	Fx length, proximal bone	0.864	Fx surface	0.000 *
	Fx length, distal bone	0.880	Fx outline	0.775
	Ratio of length, proximal bone	0.587	Type of Fx	0.469
	Ratio of length, distal bone	0.977	Fx category	0.331
	Fx angle	0.280	Location on bone	1.000
	Change in Fx length (prox. vs. dist.)	0.031 *	Fx margin	0.000 *
	Change in ratio of length (prox. vs. dist.)	0.190		
*Post-Burning vs. Post-BFI* Groups A vs. B	Fx angle	0.038 *	Fx surface	1.000
			Fx outline	0.026 *
			Type of Fx	0.000 *
			Fx category	0.009 *
			Location on bone	0.049 *
			No. of fragments	0.076
			Fragment size	0.111
*Post-BFI vs. post-BFI & burning* Groups B vs. C	Fx Length, Proximal bone	0.181	Fx surface	0.000 *
	Fx Length, Distal bone	0.027 *	Fx outline	0.272
	Ratio of length, Proximal bone	0.245	Type of Fx	0.025 *
	Ratio of length, Distal bone	0.667	Fx category	0.265
	Fx angle	0.009 *	Location on bone	0.051 *
*Burning temperature; number & class of fractures:* *Epiphyseal vs. intermediate regions, and subsequently tested specific for BFI, HIBF, iHIBF*	Group A	0.034 *	Group A – HIBF	0.390
			Group A – iHIBF	0.436
	Group C	0.040 *	Group C – BFI	0.040 *
			Group C – HIBF	0.122
			Group C – iHIBF	0.302

#### Post-BFI

Based on ANOVA analysis, group control-BFI (B) showed significantly greater angles of fractures than group BFI and burn (C) pre-burning. KW-test of categorical features between these groups showed statistical differences for the type of fracture and the location on bone; group B showed predominantly oblique fractures and had more fractures at the intermediate region than group C.

#### Post-burning

KW-test comparing groups control-burn (A), and BFI and burn (C) resulted in a significant difference for fracture surface, fracture outline, fracture category, and location on bone. Group A showed smooth fracture surface, more bones with transverse fracture outline, and more complete-simple fractures while group C showed more fractures at the intermediate region.

#### Pre- versus post-burning

Comparing unburned group BFI and burn (C) bones with burned group C bones, ANOVA and KW-test resulted in significant statistical difference only for fracture surface and sharpness of the margins. Burned group C showed rough fracture surface and loss of sharpness (blunt margins), unlike unburned bones. MW-test of the change in lengths of group C bones at the proximal and distal ends (from unburned to burned) showed a significant decrease in length of burned bones, whereas the MW test of the ratios of length on tension side to length on compression side of proximal and distal ends of unburned and burned bones did not show any significance.

#### Post-burning versus post-BFI

ANOVA and KW-test of group control-burn (A) and group control-BFI (B) showed statistical significant differences for fracture angle, fracture outline, fracture type, facture category, and location on bone. Group A displayed greater fracture angles, more fractures with transverse outline, and more complete-simple fractures than group B, and group A also showed less fractures at the intermediate region than group B.

#### Post-BFI versus post-BFI and burning

KW-test comparing group control-BFI (B) and group BFI and burn (C) showed statistical significance for length of fracture (distal), fracture angle, fracture surface, and type of fracture obtained. Group B bones exhibited greater length of fracture (distal), greater fracture angles, smoother fracture surface, and more oblique fractures.

#### Burning temperature: number and class of fractures

Group control-burn (A) showed statistical significant differences between the epiphyseal region and the intermediate region, based on MW-test, with a greater number of fractures in the intermediate region. Group BFI and burn (C) also showed significant statistical difference between the epiphyseal region and the intermediate region, with a greater number of fractures in the intermediate region. The intermediate region of group C showed significantly more post-mortem (pre-fire) fractures than the epiphyseal region of group C, based on KW-test for number of each fracture class (HIBF, iHIBF, or post-mortem pre-fire). Group A did not show this significant difference.

## Discussion

The goal of this study was to determine features distinguishing post-mortem fractures (pre-fire) reflecting peri-mortem trauma from HIBFs and iHIBFs, by using a checklist, shown in Fig. S3a and S3b in ESM Section 1, to analyze the fractures. The data collection sheet was mainly derived from literature. The data collection sheet made fracture analysis, and thereby distinguishing features of HIBFs from BFI-fractures, more objective and efficient by combining qualitative and quantitative characteristics which in turn allowed more information to be extracted from burned bones. The data collection sheets from this study need to be tested in more anthropological studies to accurately evaluate them for their usability, precision, validity, and eventual implementation in casework. Inter-observer errors, as well as false positive/negatives, should be further explored with follow-up blind studies.

Casework-relevant conditions were mimicked using human material, burning bones surrounded by skin, and clothing within a room resembling a living room and allowing forearm bones to be surrounded by air and flaming combustion. The latter is similar to forearms of a body that takes on a pugilistic posture during a fire, whereby the rapid dehydration of muscular tissues and strong muscle contraction cause forearms to move away from the torso, allowing increased fire and oxygen exposure around them [24, 25]. Blunt force trauma to the human forearms, mimicking self-defense mechanism, and calcined remains are both on their own common in forensic contexts [3]. Some of the factors affecting fracture propagation and burning were standardized to understand the fracture features and any patterns observed, and to account for some confounding factors. These factors included defleshing and refleshing to remove the variability between humans in terms of thickness of skin, adipose, and muscle tissue. However, it is crucial to note that the bone to muscle attachments (tendons) were also removed in this process, thereby removing the structure that can result in specific fractures due to mechanical stress. By reducing the variability of the tissues, the force of blunt-impact and thermal stress on the bone were more equalized. The chosen parameters were based on past studies and theoretical knowledge. The usage of defleshed forearm bones, controlled burning conditions, and investigating complete fractures only, albeit casework-relevant, are not reflective of all casework circumstances [26].

One of the variables present in the sample that can influence the outcome is variation in bone mineral density (BMD) [10, 27, 28]. All used bones were extracted from individuals in the old adult age group (50 + years, based on [22]), which are more mineralized causing increased brittleness, and thus being more susceptible to fracturing. Since BMD was not assessed in this study, osteopenia and osteoporosis cannot be completely excluded, despite efforts taken to exclude osteoporotic samples. Such variation can affect the spread of data and subsequently statistical results.

Since the process of bone fracturing is a multifactorial processes with many influencing factors, it is hard to replicate exact casework conditions in the laboratory and conversely, to apply scientific interpretation to casework. For this study, most importantly, the average heat exposure was estimated to be equal amongst the heated groups, which allows for a comparison between these groups on the chosen features. However, despite the intended equalization in fire dynamics, with a measured temperature range of 700 to 800 °C at the flaming outskirts for the majority of the duration, the burned bones did show differences in exposure temperature. Furthermore, the estimated exposure temperature was lower than the measured temperature range of the fire. Flame temperature was not measured at the location of the pyre but instead at the perimeter. Lower temperatures can be expected at the base of the flame due to movement of air and endothermic processes, and the temperature at the tip of the flames is higher than at the base due to fire dynamics [29], explaining the lower estimated temperature of the bones compared to the temperature of the flames. Furthermore, variation in external variables can explain local temperature differences resulting in difference in heat-induced changes of bones; most importantly, drafts influence the growth and path of the fire; this is similar to real house fire. These differences can explain the variation in estimated exposure temperature, based on colorimetric measurements, even within a single bone.

The control-BFI (B) group showed different fracture features than BFI and burn (C) pre-burning; the variation within these features was too large; this hampered the analysis on specificity of these features for the current study. The statistical differences (for fracture angle, fracture type, and location on bone) between group B (and group C, prior to burning) could be attributed to the varying distribution of bone dimensions (weight and length, see the box plots within Fig. S9 in ESM Section 4) and possible differences in BMD of each bone in either group. Therefore, statistically significant differences for fracture angle and type of fracture of subsequent analyses were not taken in to account for the conclusion. These features should be studied in a larger sample size. The significant difference found for fracture angle, and outline, between BFI and (i)HIBFs is considered to be non-representative for casework due to the possible larger variation in fracture angles in non-standardized, more close to real life, situations. The determination of fracture angles using photography and measurement tools prevents imprecise measurements, such as from using a protractor on the bones that naturally tend to curve slightly on one side. Errors are critical in scientific research; the main ones associated with this study were observer and measurement errors. Observer errors are difficult to measure due to complexity of features observed and were not statistically evaluated. Measurement error was investigated only for the angle of fractures since the angles were estimated using ImageJ. A relatively low value of 1.55° was obtained for the average standard error (for all bones) (Table S21 in ESM Section 9), which substantiates the reliability of the results from the study.

Both control-burn (A) and BFI and burn (C) showed more fractures in the intermediate section of the bone, while, as expected, group C showed more fractures in that region when compared to group A. The intermediate section apparently has a higher chance of fracturing as a result of heat than the epiphyseal ends. This increases the chance on a false-positive finding if BFI-specific features are not sufficiently available for differentiation. Fragmentation (number and size) was inspected in this study, but in real situations, fragmentation of burned bones is difficult to accurately determine and is likely to increase from post-fire handling. This adds on to the forensic relevance of this study as fragmentation can be common in casework and the approach from this study can be possibly used in these cases. Some fragments might be missing or lost in the fire debris; thus, fragmentation number and size are not usable as parameters for the fracture distinction—fragmentation pattern would be a better observation.

The results on the features from post-mortem (pre-fire) fractures agreed with literature—slanted, sharp fracture margins, smooth surface, and no heat-induced color change of fracture margin with respect to rest of the bone. The fracture angles of these fractures were rather parallel to the longitudinal axis of bone than being perpendicular, agreeable to the results of Ioana et al. [7]. The majority of the post-mortem (pre-fire) fractures from this study were classified as oblique fractures, although the fracture outlines were transverse or not an exact diagonal. This classification stems from Wedel and Galloway’s descriptions of three variations of oblique fractures [9], based on how the combined forces of angulation and compression interact—purely oblique (failure in compression), oblique resembling transverse fracture (large bending forces), and oblique-transverse (starts as transverse with tension and compression acting on remaining bone). This observation emphasizes the importance of understanding the variations within single fracture type and ensuing diversity in fracture features before examining traumatic fractures in bones, to avoid misidentification.

Most post-fire results obtained from group C (BFI and burn) are consistent with previous studies, for example, the observed difference between group A (control-burn) and group C in fracture surface morphology—smoother for HIBFs and rougher for post-mortem (pre-fire) fractures [1, 4, 8, 30]. However, the observed smoothness in sloped/curved parts of fracture surface at the margins and roughness in remaining part has not, to our knowledge, been mentioned before. This coexistence of fracture surface types could arise from uneven heat distribution between a flat area as opposed to a curved area. Another probable reason would be the coexistence being a by-product of the ongoing heat-induced changes in the bone such as transitions in different phases of burning—for example, dehydration and loss of organic constituents to conversion and crystallization of inorganic elements [11, 31]. Herrmann et al. described trouble in interpreting longitudinal fractures in burned bones since such fracture propagation is associated with both burning and trauma [1]. Most of the traumatic fractures before burning (post-mortem pre-fire, of the study of Hermann et al.) were longitudinal, whereas no longitudinal post-mortem pre-fire fractures were found in the present study and only longitudinal HIBFs were observed [1]. Thus, it was easier to identify the longitudinal HIBFs in this study. This highlights the influence of post-mortem (pre-fire) fracture type on the difficulty of differentiating these fractures from HIBFs and iHIBFs.

An interesting finding of this study was the elevation of one side of the iHIBFs, and that this feature was not found when examining HIBFs. This finding is diametrically opposed to the findings of Bohnert et al. (1998), who referred to it as rolled up edges, and associated their finding with HIBFs [32]. That means that there is no consensus yet about this feature, and it is not useable for differentiation between different fracture classes. The one-sided elevation was not caused by incorrect handling of the burned remains since it was observed prior to removing the remains from the burning location. The feature was even observable when the longitudinal fracture halted and continued past the BFI, as was observed in reconstructed diaphyseal sections. Possibly, the longitudinal fracture originated on both sides from the BFI site due to the presence of microfractures. Sudden temperature drops during the end-phase of burning and subsequent extinguishing of fire using water could have caused this difference in elevation per side. This hypothesis requires more research. This is especially important since extinguishing fire with water is a common practice and has not gained much attention in prior studies [33]. Alternatively, the elevation could have resulted from differences in shrinkage of bone on either side as a type of warping, due to the differential burning of the bone [4, 24, 34].

Warping was seen in only few bones that were closer to complete calcination (temperature range of 450–900 °C), where these regions probably received more heat. Warping has been associated with fleshed bones and detected much less in defleshed bones [4, 17, 24, 35]. The dehydration and shrinkage of the bones caused small cracks in the bones. Some bones showed superficial cracking (*craquelle*) while others were more depressed. The depressed cracking could be mistaken for a pre-fire fracture if whole burned bones were found in a crime scene, leading to erroneous analyses. Post burning, the BFI-fractures and HIBFs showed more calcination and less carbonization at the fracture margins and surface than iHIBFs, which could arise from an effect of the lesser duration- or lack-, of heat exposure. In the case of prolonged exposure, this feature can be expected to diminish.

Only the anterior portion of the bone was utilized for colorimetric analysis due to high brittleness and post-recovery breakage of the bones. Therefore, the colorimetric analysis only represents the parts of the bone that were, most likely, exposed to the highest temperature since the posterior portion was partly shielded from the heat by the bone itself, assuming the bones did not rotate during the burning process. Upon observing a greater number of fractures at the intermediate region, where calcination was also more often observed, it can be deduced that this region received most heat during burning and was exposed more to the dynamics of the fire, which could be due to the more central positioning of the bones in the fire. Moreover, the structure of bone differs between the intermediate and epiphyseal regions, and thus, heat-induced changes related to temperature differ for these 2 structures.

There is a need for more empirical research to investigate crucial influential factors in relation to thermal exposure of bodies to strengthen the interpretation of fractures in burned bone. Such factors include BMD levels in healthy and diseased individuals, age, lifestyle and physiological changes, presence of soft tissue, and their effect on fracture (both traumatic and heat-induced) propagation. Due to the numerous factors affecting the fracture patterns, an individualistic and standardized approach cannot be used for differentiation of traumatic fractures from heat-induced fractures. Instead, a combination of analytical techniques and/or more quantitative methods such as chemical, physical analyses, and 3D imaging (like digital microscope, micro-CT) needs to be investigated to enable reproducible and accurate results [2].

## Conclusion

The descriptive and quantitative data obtained showed that the checklist used in this study allows for a more objective, highly systematic, and comprehensive analyses of burned bones.

The colorimetric analysis resulted in an equal estimation of heat exposure for both the control and burn group and the group BFI and burn; this justifies a comparison between the two heated groups. Out of all statistical analysis, the features fracture surface and fracture margin showed to be most discriminating between the three studied groups: control and burn (expected to contain heat induced bone fractures, HIBF, and indirect heat induced bone fractures, iHIBF), control blunt force impact fracture without burning (containing post-mortem non-traumatic bone fractures, nTBFs), and lastly the group that was burned after BFI (expected to containing all possible fracture classes). Significant differences were found between the morphology of the fracture surface and margin, and notable differences between discoloration of the fracture margin and surface between fracture classes post-mortem (pre-fire) and HIBFs or iHIBFs. However, the findings on these features were comparable between HIBFs and iHIBFs, hampering further differentiation. These fracture classes are therefore useful to support conclusions on hypothesis that requires differentiation between fractures present before the fire and those that occurred during or after the fire. Furthermore, overlapping features between fracture classes post-mortem (pre-fire), HIBFs and iHIBFs were also identified; these features are therefore not useful for differentiation.

The limitations of this study largely stem from the controlled experimental conditions, which might not be applicable to all casework. The interpretation of fractured bones salvaged from a burning context is hampered by an array of internal and external influential factors. An approach using common case-relevant factors and involving a combination of computerized and analytical techniques with lesser observer-based involvement would facilitate more accurate interpretation.

## Supplementary Information

Below is the link to the electronic supplementary material.

Supplementary file1 (DOCX 16628 KB)

## Abbreviations

HIBF: Heat-induced bone fracture
iHIBF: Indirect heat-induced bone fracture
HI: Heat-induced
BFI: Blunt force impact
nTBF: Non-traumatic bone fracture
