# Supplementary material for: Mechanical or thermal damage: differentiating between underlying mechanisms as a cause of bone fractures
Source: Int J Legal Med. 2022 Apr 30;136(4):1133–48. doi: 10.1007/s00414-022-02825-x (PMC9170644; doi:10.1007/s00414-022-02825-x)
Supplement: Supplementary file 1 — Supplementary file1 (DOCX 16628 KB) [file 414_2022_2825_MOESM1_ESM.docx]

**Mechanical or thermal damage, differentiating between underlying mechanisms as a cause of bone fractures.**

Divya S ^1, *^, Tristan Krap ^2,3,4, *^, Wilma Duijst ^3,4^, Maurice C.G. Aalders ^1^, Roelof-Jan Oostra ^2^,

1. Amsterdam UMC, Location AMC, department of Biomedical Engineering and Physics, The Netherlands
2. Amsterdam UMC, Location AMC, department of Medical Biology, section Clinical Anatomy and Embryology, Amsterdam, The Netherlands
3. Maastricht University, Maastricht, The Netherlands
4. Ars Cogniscendi Foundation for Legal and Forensic Medicine, Wezep, The Netherlands

*. Shared first authorship

Corresponding author: Tristan Krap

Mail: [t.krap@amsterdamumc.nl](mailto:t.krap@amsterdamumc.nl)

Address: Amsterdam UMC, Location AMC,
department of Medical Biology, section Anatomy
Meibergdreef 15, 1105 AZ Amsterdam, The Netherlands

# Electronic Supplement Material (ESM) section 1 – Existing literature and checklist derived from former studies

| **Feature of fracture(s)** | **Studies which focused on respective features** |
| --- | --- |
| Degree/State of burning of bone | *Mayne Correia, 1997; Herrmann & Bennett, 1999; Krap, van de Goot, Oostra, Duijst & Waters-Rist, 2017; Macoveciuc, Márquez-Grant, Horsfall & Zioupos, 2017* |
| Fragmentation | *Herrmann & Bennett, 1999; Macoveciuc, Márquez-Grant, Horsfall & Zioupos, 2017* |
| Colour of bone/fragments | *Mayne Correia, 1997; Herrmann & Bennett, 1999* |
| Fracture category | *Macoveciuc, Márquez-Grant, Horsfall & Zioupos, 2017* |
| Location of fracture on bone | *Mayne Correia, 1997; Herrmann & Bennett, 1999; Macoveciuc, Márquez-Grant, Horsfall & Zioupos, 2017* |
| Fracture outline, surface morphology and angle | *Villa & Mahieu, 1991; Mayne Correia, 1997; Herrmann & Bennett, 1999; Outram, 2004; Wieberg & Wescott, 2008; Wheatley, 2008; Poppa et al., 2011; Macoveciuc, Márquez-Grant, Horsfall & Zioupos, 2017* |
| Dimensions of fracture and bone | *Thompson, 2005; Waltenberger & Schutkowski, 2017* |

**Table S1**: The various features investigated by previous studies and corresponding authors. The various features employed in current study and the corresponding former studies, which investigated fractures using these features. The degree/state of burning and colour of bone account for changes in bone such as fractures caused by exposure to burning/heat. This list of features is not exhaustive. There are other features as well but those mentioned are the ones more commonly used for studies involving trauma analysis and/or thermal alterations.

| **Study** | **Material, size (N) & grouping** | **Burning method** | **Analysis method** | **Results** |
| --- | --- | --- | --- | --- |
| *Herrmann & Bennett, 1999 [1]* | Femora of domestic pig, *Sus scrofa*, with minimal soft tissue and articulated patellae;  N= 41;  12 sharp, 8 gunshot, 8 blunt force trauma, 5 torsional loading & 8 controls. | 1) Frame house, initiated with accelerant (groups: sharp, gunshot, blunt and controls).  2) Firebox for intense wood fire (group: torsional loading). | Three randomly selected fragments from each sample were visually assessed: degree of burning, fragmentation, fracture patterns, facture surface morphology magnified. Followed by microscopic assessment by a stereomicroscope at 35-70X magnification, & SEM. | - Degree of burning: majority was calcined. - Fractures exhibited characteristics that reflected the mode of fracturing: burning, situational or traumatic. - Characteristics of sharp force trauma remained evident post-burning. - Characteristics of gunshot trauma could not be distinguished post-burning due to fragmentation. - Characteristics of blunt force were less specific post-burning. - Traumatic and heat-induced fractures exhibited similar characteristics, especially the surfaces of longitudinal fractures. - Situational fractures (iHIBFs) were the easiest to distinguish. |
| *de Gruchy & Rogers, 2002 [16]* | Pig radii and ulna (frozen with 1cm of muscle around bones) & beef ribs (fleshed);  N=30 forelimbs and 30 ribs;  5 cleaver and knife marks on each bone. | Outdoor fire, duration of 3 hours,  contained within a ring of steel, mix of soft and hardwood & no accelerants. | Samples were sorted based on visible trauma, lesions were assessed at1.6X magnification by means of a stereomicroscope. | - Chop mark features of forelimbs were mostly unaffected by burning. - Only notable difference between chop marks observed on fresh and burnt bone was the size of the roughened point of exit, which increased when exposed to fire. - No conclusive chop marks were observed on the ribs due to the almost complete destruction of these elements by fire. |
| *Pope & Smith, 2004 [4]* | Unembalmed human heads;  N=40;  16 ballistic force, 8 blunt, 6 sharp and 10 non-traumatized heads (intact bodies) as controls. | Open-air fire with combinations of wood, metal reflectors, charcoal, & accelerants. | Visual macroscopic morphological examination, photographed using tangential lighting, and microscopically assessed at 10-40X magnification by means of an operating  microscope. | - Distinct characteristics of ballistic, blunt, and sharp force trauma survived varying degrees of thermal degradation. - Analysis of ballistic and blunt force trauma after burning was complicated by delamination and fragmentation of primary trauma sites due to heat exposure. |
| *Poppa et al., 2011 [18]* | Pig heads;  N=9;  3 sharp force lesion, 3 blunt and 3 gunshot. | Iron grill and 2 gas  cookers. Burning process divided in to 4 phases. | Analysis was carried out each burning phase; lesions were photographed and investigated with a stereomicroscope. | - Morphological characteristics of lesions, produced by different tools, were obscured by carbonization. - These characteristics became visible again in the last stage of the burning process, due to loss of carbonized soft tissues and calcination of bone. |
| *Macoveciuc et al., 2017 [8]* | Fresh juvenile sheep radii under 3 years of age & defleshed later;  N=20;  2 to determine optimal experimental conditions, 14 for  the applied mechanical trauma and 4 controls | Closed-compartment “fire” reproduced in an electric furnace. | Visual macroscopic, morphological examination of each specimen & before-after fire comparison by using photographs. | - Specific sharp and blunt force trauma characteristics were not completely masked by heat-induced changes. - HIBFs were not obscured by post-cremation breakage. - iHIBFs only occurred along pre-existing penetrating heat fractures, aggravated superficial fracture lines and originated from the point of mechanical trauma impact. |

* *Table is not meant to be exhaustive, it presents a selection of literature, including literature that was used for the development of the data collection sheets, see section “data collection via macroscopic and microscopic analysis” within the “materials and methods” section and fig.s3a and s3b.*

***Table S2****:* *Key findings of previous studies on the effect of heat on fracture analysis, including sample material, methodology.*

**CHARACTERISTICS (BFT)**

| **SPECIMEN NUMBER** |  |
| --- | --- |
| **TYPE OF BONE** | Radius / Ulna |
| **TYPE OF SPECIMEN** | Group A (control) / Group B / Group C |

| **MACROSCOPIC OBSERVATIONS** | | | | **REMARKS** |
| --- | --- | --- | --- | --- |
| **Number of fractures** | - 1 - 2-5 - >5 | | |  |
| **Fragmentation**  *(Herrmann & Bennett, 1999)* | - Yes - No | | | - Number of fragments: - Size of fragments:   Small (<3 cm), Medium (3–5 cm) or Large (>5 cm) |
| **Colour of bone/fragments**  *(Herrmann & Bennett, 1999)* | - Unaltered - Brown - Black - Gray - Gray-blue - White | | |  |
| **Fracture category**  *(Macoveciuc, Márquez-Grant, Horsfall & Zioupos, 2017)* | - Complete-simple (2 separate pieces of bone) - Complete-comminuted (≥ 2 separate pieces of bone) - Incomplete (pieces of bone still joined together) | | |  |
| **Location of fracture on bone** *(Herrmann & Bennett, 1999)* | - Proximal - Intermediate - Distal | | |  |
| **Fracture outline**  *(K. Outram, 2002)* | - Helical/ curved - Transverse (75° - 105°) - Longitudinal and transverse - Diagonal - Diagonal with a step   Columnar | - Sharp - Blunt | - Clearly defined - Not clearly defined |  |
| **Fracture surface morphology**  *(Wheatley, 2008) (Wieberg & Wescott, 2008) (Herrmann & Bennett, 1999) (Villa & Mahieu, 1991)* | - Smooth (even and fine texture) - Rough (uneven or bumpy/irregular texture) | | |  |

| **MICROSCOPIC OBSERVATIONS** | | | **REMARKS** |
| --- | --- | --- | --- |
| **Dimensions of BFT fracture**  *(Thompson, 2005)* | **Proximal** | **Distal** | (Fracture dimensions will be based on compression and tension sides) |
|  | - C: - T: | - C: - T: |  |
| **Fracture angle (wrt longitudinal axis of bone)**  *(Herrmann & Bennett, 1999) (Villa & Mahieu, 1991) (K. Outram, 2002)* | - Right angle - Acute angle (less than 90°) - Obtuse angle (wider than 90° and less than 180°) | |  |

| **OVERALL CONCLUSIONS** | |
| --- | --- |
| **Type of traumatic fracture:** | **Extra notes:** |

***Fig. S3a****: Checklist of features used to examine the fractures along with the studies they were derived from for post-BFT analysis.*

**CHECKLIST FOR FRACTURE CHARACTERISTICS (BURNING)**

| **SPECIMEN NUMBER** |  |
| --- | --- |
| **TYPE OF BONE** | Radius / Ulna |
| **TYPE OF SPECIMEN** | Group A (control) / Group B / Group C |

| **MACROSCOPIC OBSERVATIONS** | | | | **REMARKS** |
| --- | --- | --- | --- | --- |
| **Number of fractures** | - 1 - 2-5 - >5 | | | (label each fracture with location on bone) |
| **Degree/State of burning of bone**  *(Mayne Correia & Beattie, 2001) (Herrmann & Bennett, 1999)* | - Unmodified - Carbonized - Partially burnt - Calcined - Complete (ashes) | | |  |
| **Fragmentation**  *(Herrmann & Bennett, 1999)* | - Yes - No | | | - Number of fragments: - Size of fragments:   Small (<3 cm), Medium (3–5 cm) or Large (>5 cm) |
| **Colour of bone/fragments**  *(Herrmann & Bennett, 1999)* | - Unaltered - Brown - Black - Gray - Gray-blue - White | | |  |
| **Fracture category**  *(Macoveciuc, Márquez-Grant, Horsfall & Zioupos, 2017)* | - Complete-simple (2 separate pieces of bone) - Complete-comminuted (≥ 2 separate pieces of bone) - Incomplete (pieces of bone still joined together) | | |  |
| **Location of fracture on bone** *(Herrmann & Bennett, 1999)* | - Proximal - Intermediate - Distal | | |  |
| **Fracture outline**  *(K. Outram, 2002)* | - Helical/ curved - Transverse (75° - 105°) - Longitudinal and transverse - Diagonal - Diagonal with a step   Columnar | - Sharp - Blunt | - Clearly defined - Not clearly defined |  |
| **Fracture surface morphology**  *(Wheatley, 2008) (Wieberg & Wescott, 2008) (Herrmann & Bennett, 1999) (Villa & Mahieu, 1991)* | - Smooth (even and fine texture) - Rough (uneven or bumpy/irregular texture) | | |  |

| **MICROSCOPIC OBSERVATIONS** | | | **REMARKS** |
| --- | --- | --- | --- |
| **Dimensions of BFT-fracture/ bone length**  *(Thompson, 2005)* | **Proximal** | **Distal** | (Fracture dimensions will be based on compression and tension sides) |
|  | - C: - T: | - C: - T: |  |
| **Fracture angle (wrt longitudinal axis of bone)**  *(Herrmann & Bennett, 1999) (Villa & Mahieu, 1991) (K. Outram, 2002)* | - Right angle - Acute angle (less than 90°) - Obtuse angle (wider than 90° and less than 180°) | |  |

| **OVERALL CONCLUSIONS** | |
| --- | --- |
| **Type of traumatic fracture:** | **Type of heat-induced fracture:** |
| **Heat-induced fracture:** | **Type of fracture:** |

***Fig. S3b****: Checklist of features used to examine the fractures along with the studies they were derived from for post-burning analysis*

# ESM section 2 – Data about sample material

| **Label No.** | **Sex** | **Age** | **Weight (g)** | **Maximum length (cm)** | **Circumference (cm)** | **A-P width (cm)** |
| --- | --- | --- | --- | --- | --- | --- |
| 1313R-Lt | F | 64 | 70 | 23.8 | 5 | 1.8 |
| 1313U-Lt | F | 64 | 84 | 26.8 | 4.8 | 1.6 |
| 06217R-Rt | F | 71 | 71 | 25.5 | 4.9 | 2.2 |
| 06217U-Rt | F | 71 | 81 | 27.3 | 4.7 | 1.6 |
| 06217R-Lt | F | 71 | 70 | 25.4 | 4.7 | 1.6 |
| 06217U-Lt | F | 71 | 80 | 26.9 | 4.9 | 1.7 |
| 14917R-Lt | F | 72 | 55 | 24.3 | 4.4 | 1.5 |
| 14917U-Lt | F | 72 | 65 | 25.9 | 4.2 | 1.4 |
| 08017R-Rt | F | 74 | 65 | 23.6 | 4.9 | 1.7 |
| 08017U-Rt | F | 74 | 79 | 25.5 | 4.9 | 1.8 |
| 08017R-Lt | F | 74 | 61 | 23.8 | 4.5 | 1.5 |
| 08017U-Lt | F | 74 | 74 | 25.6 | 4.8 | 1.6 |
| 10817R-Rt | F | 76 | 54 | 22.6 | 4.2 | 1.3 |
| 10817U-Rt | F | 76 | 69 | 24.8 | 4.7 | 1.5 |
| 10817R-Lt | F | 76 | 54 | 22.9 | 4.3 | 1.6 |
| 10817U-Lt | F | 76 | 70 | 25.1 | 4.8 | 1.7 |
| 09317R-Lt | F | 77 | 69 | 23.3 | 5 | 1.9 |
| 09317U-Lt | F | 77 | 79 | 25.2 | 5.3 | 2 |
| 09317R-Rt | F | 77 | 73 | 23.6 | 5.1 | 1.7 |
| 09317U-Rt | F | 77 | 83 | 25.9 | 5.2 | 1.8 |
| 04018R-Lt | F | 84 | 53 | 22.7 | 4.5 | 1.6 |
| 04018U-Lt | F | 84 | 66 | 24 | 4.7 | 1.7 |
| 04018R-Rt | F | 84 | 55 | 22.8 | 4.3 | 1.6 |
| 04018U-Rt | F | 84 | 63 | 23.8 | 4.7 | 1.6 |
| 02117R-Rt | F | 86 | 94 | 25.7 | 5.3 | 2 |
| 02117U-Rt | F | 86 | 110 | 27.8 | 5.3 | 2 |
| 02117R-Lt | F | 86 | 90 | 25.5 | 5.4 | 2.1 |
| 02117U-Lt | F | 86 | 111 | 27.5 | 5.7 | 2.1 |
| 12117R-Lt | M | 66 | 84 | 25.6 | 5.2 | 1.7 |
| 12117U-Lt | M | 66 | 106 | 28.2 | 5.4 | 2 |
| 12117R-Rt | M | 66 | 87 | 25.8 | 5.2 | 1.7 |
| 12117U-Rt | M | 66 | 105 | 27.4 | 4.8 | 1.6 |
| 18017R-Lt | M | 84 | 74 | 25.4 | 5.3 | 2.1 |
| 18017U-Lt | M | 84 | 88 | 27.3 | 5.3 | 1.9 |
| 18017R-Rt | M | 84 | 70 | 25.5 | 5.1 | 1.7 |
| 18017U-Rt | M | 84 | 85 | 26.9 | 5.3 | 1.8 |
| 5313R-Lt | M | 88 | 69 | 25.3 | 4.9 | 1.7 |
| 5313U-Lt | M | 88 | 81 | 26.6 | 5.1 | 2.1 |

***Table S4****: Population data and dimensions of the de-fleshed cadaveric bones.* ***R****: Radius,* ***U****: Ulna,* ***Rt****: bone from right arm,* ***Lt****: bone from left arm. Green shaded rows denote the bones used to make sections for the burning-pilot study. Orange shaded rows denote the bones used for BFT-fracture production-pilot study. *****Maximum length of radius****: distance between styloid process and the proximal extremity of radial head;* ***maximum length of ulna****: distance between styloid process and the proximal extremity of olecranon.*

# ESM section 3 – Pilot studies to determine standard conditions

***(i) BFI fracture generation***

BFI fractures were created on 5 fresh-frozen, defleshed bones by the custom-made contraption, resembling a pendulum apparatus (Fig. 1 of the manuscript). The grading element (0° to 180°) indicates the angle at which the rod was released. To determine the ideal angle needed to produce the intended fractures, the rod was released from various angles till the bones showed similar fractures. The impact area of the rod on the bone was visualized using Pelikan green ink – label sticker was placed around the cylindrical impactor and dabbed with the ink. The rod was slowly released onto the bone till the ink transferred to the bone, representing the impact area. The impact energy, area and force needed to generate the fractures were then calculated using formulae presented below in Box S5.

***Box S5****: Calculations for the impact area and impact force, whereby all measurements are in respective SI units.* ***Legend****:* ***a*** *– horizontal radius of ellipse,* ***b*** *– vertical radius of ellipse,* ***m*** *– mass of rod,* ***v*** *– velocity,* ***g*** *- acceleration due to gravity (9.8m/s^2^ on Earth),* ***h*** *- height of rod,* ***t*** *– time of contact between rod and bone during impact (in this study, the time ranges from 0.009s to 0.004s).*

*Using dimensions of the elliptical impact area,*

$$\boldsymbol{Area of impact, A}=\pi ab=\pi\times\left( 0.007\div2 \right)\times\left( 0.003\div2 \right)=\boldsymbol{1.65\times}\boldsymbol{10}^{\boldsymbol{-5}}\boldsymbol{m}^{\boldsymbol{2}}$$

*By the conservation of energy,*

$Potential energy of rod=Kinetic energy at impact on bone$*,*

$mgh= \frac{1}{2}mv^{2}$

$$v= \sqrt{\frac{\left( 9.8\times0.49 \right)}{\left( \frac{1}{2} \right)}}\approx3.10 m/s (to 2 decimal places)$$

$$\boldsymbol{Impact force, F}=\frac{2mv}{t}=\frac{2\times3\times3.1}{0.004 to 0.009}=\boldsymbol{4650}\boldsymbol{N to 2066}\boldsymbol{N}$$

Table S6 below, shows the different angles used to generate fractures on the bones. Apart from 40° and 50°, which failed to produce fractures, the other angles resulted in complete fractures. The ideal angle was selected based on whether the impact will be consistent among all sample bones, despite the varying bone densities. At 130°, the impact was deemed to be too strong to be used for bones with lower densities. At 60°, the impact was deemed to be insufficient for bones with higher densities. As such, 80° was chosen to allow consistent impact across all bones and was used as the standard angle of release of rod for the main study. The area of impact on bone (depicted in 14917U-Lt of Table S6) and the impact force were calculated to be $1.65\times{10}^{-5}m^{2}$ and $2066N-4650N$ respectively.

| **Specimen** | **Angle at which rod is released from (°)** | **Observations** | **Images** |
| --- | --- | --- | --- |
| 04018U-Lt | 130 | Complete fracture with tiny fragments | 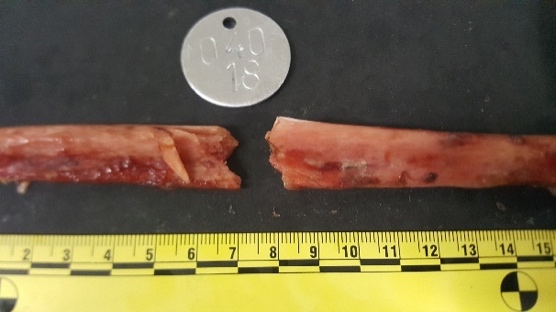 |
| 04018R-Rt | 90 | Complete fracture | 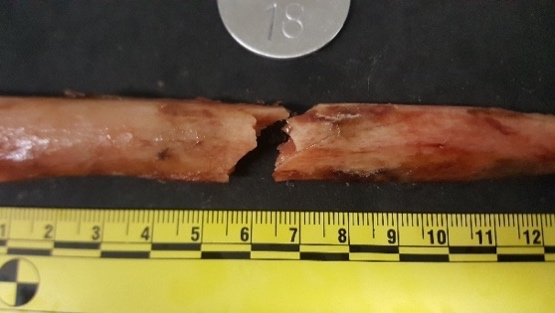 |
| 04018U-Rt | 70 | Complete fracture | 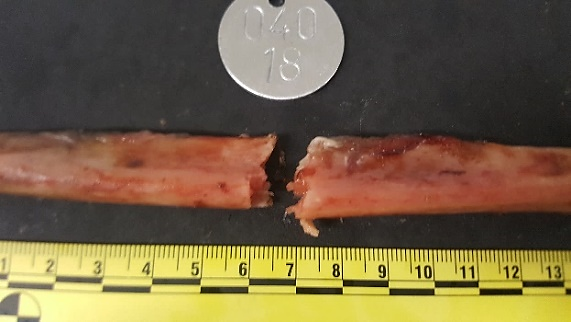 |
| 10817R-Lt | 40 | No fracture |  |
| 10817R-Lt | 50 | No fracture |  |
| 10817R-Lt | 60 | Complete fracture | 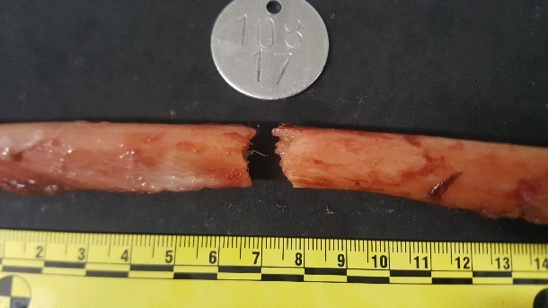 |
| 14917U-Lt | 80 * | Complete fracture | 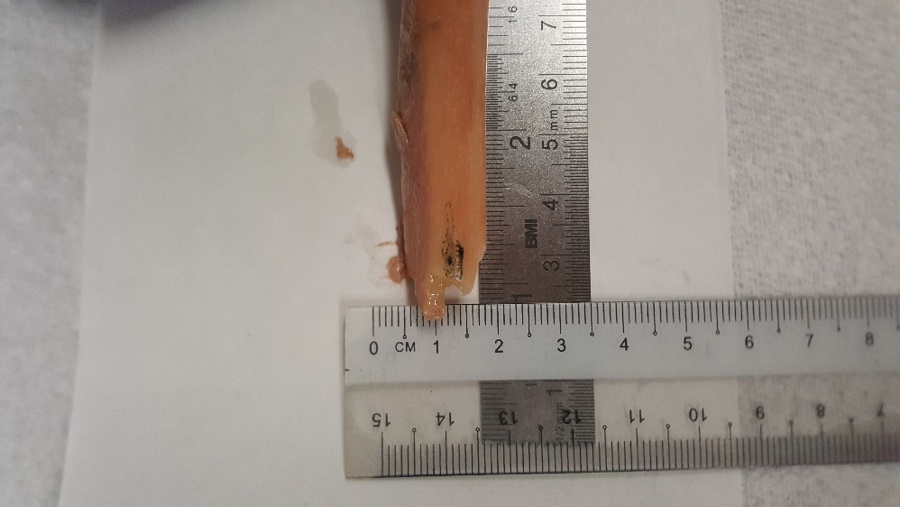 |

***Table S6****: Observations from the pilot study to identify the ideal angle for the generation of BFI fracture, which is denoted by the asterisk. 14917U-Lt shows the impact area (an ellipse) caused by the rod while fracturing the bone.*

***(ii) Burning temperature and duration***

2-5 cm transverse sections of the diaphysis of 2 radial bones were made using handsaw, whereby the bone was kept wet to avoid undesirable heat from the friction of sawing. These sections were placed in small porcelain cups were heated in a muffle oven (accuracy of ± 2°C). A temperature range of 600°C to 700°C and time intervals of 25 to 45 minutes were utilized to determine ideal temperature and duration for attaining the inversion to calcination stage of burning. Owing to the great difficulty of controlling fire temperature to accurately stop the burning at any prior stages and collect bones within stipulated time limits, this burning stage has been selected. Furthermore, the checklist was tested in this part to ensure the chosen list of features were appropriate and feasible for the remainder of the project.

Table S7 below, indicates the results obtained for determining the ideal temperature and duration for the transitionary burning stage from inversion to calcination. The temperature and duration were varied by trial-and-error method and depending on the result observed in the preceding bone-section. The highlighted sections show the most apt temperature and duration for the preferred burning stage, which can be collated as: 670°C to 690°C for temperature and 30 to 40 minutes for duration, as the standardized conditions for the main study. One bone section with the epiphysis extending into the diaphysis was also utilized to ensure if the obtained result could be replicated in a longer section of bone with slightly more soft tissue, which was successful. The burned bones were also scrutinized as per the formulated checklist. The checklist was indeed effective, whereby many features (except microscopic observations) were characterizable according to the list. Few heat-induced fractures were also seen.

| **Specimen** | **Length (cm)** | **Temperature (°C)** | **Duration of burning (mins)** | **Observations** | **Images** |
| --- | --- | --- | --- | --- | --- |
| 10817R-Rt | 4.7 | 600 | 25 | Dark grey and some grey-blue colouration with longitudinal HIF | 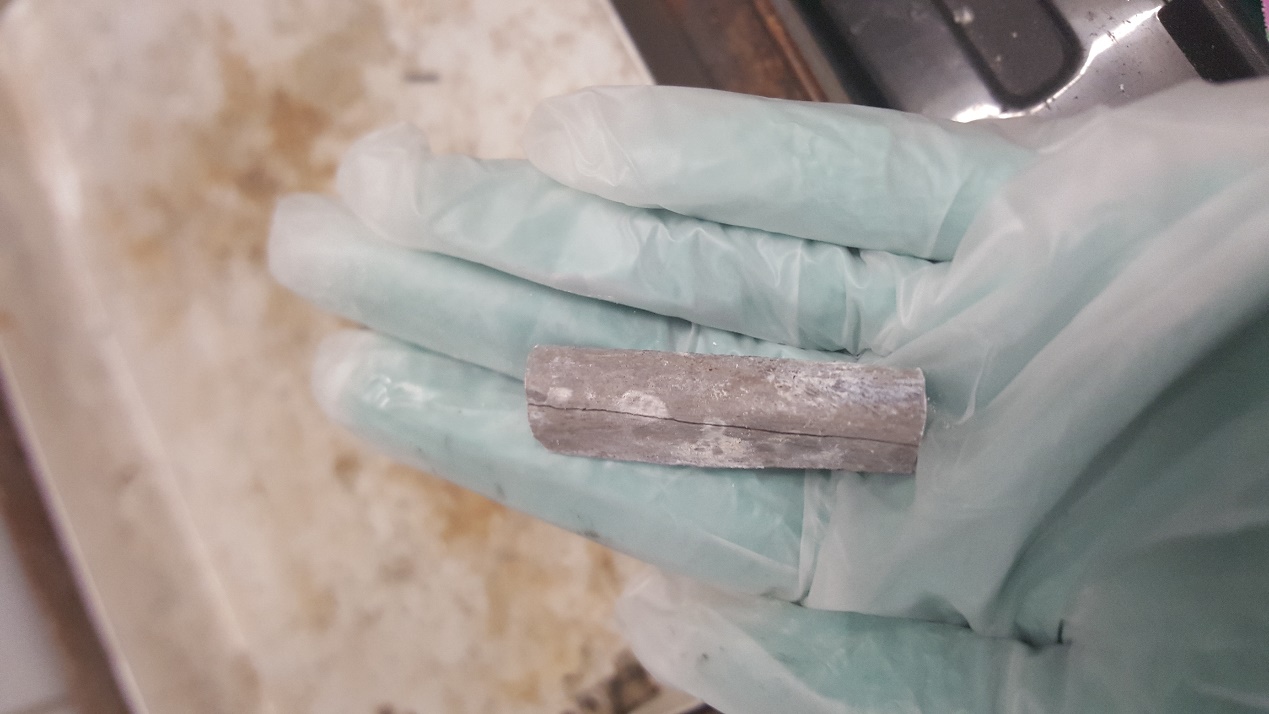 |
| 10817R-Rt | 3.9 | 620 | 30 | Mostly grey and grey-blue with some yellowish-orange regions, early longitudinal HIF | 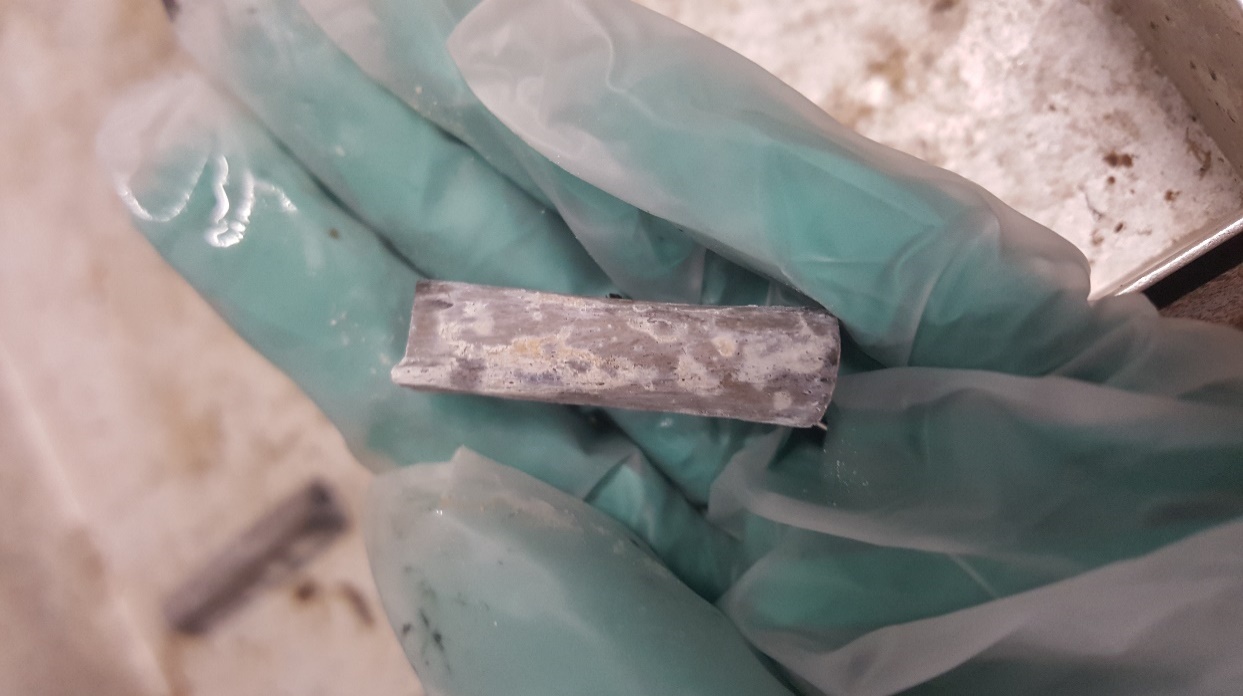 |
| 04018R-Lt | 2.2 | 650 | 25 | Mostly grey and grey-blue with patches of yellowish-orange and white regions, no HIF; medullary cavity more white than previous burned specimens | 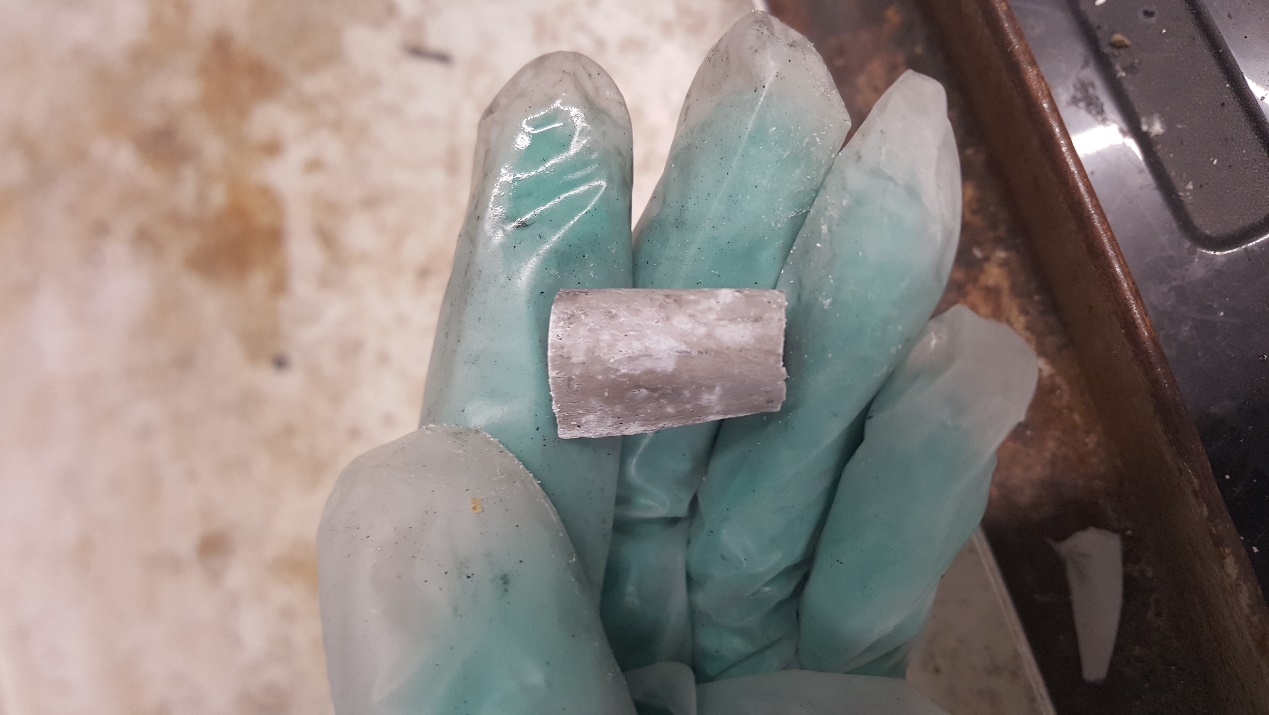 |

***Table S7 1/2****: Observations from the pilot study to identify the ideal temperature and duration of burning for the inversion to calcination stage to occur. Yellow highlighted sections denote the afore-mentioned factors apt for the desired stage of burning.*

| 04018R-Lt | 2.1 | 650 | 35 | Almost 50-60% white only on one side of bone section, less grey but more grey-blue areas, spongy layer more clumped together, no HIF | 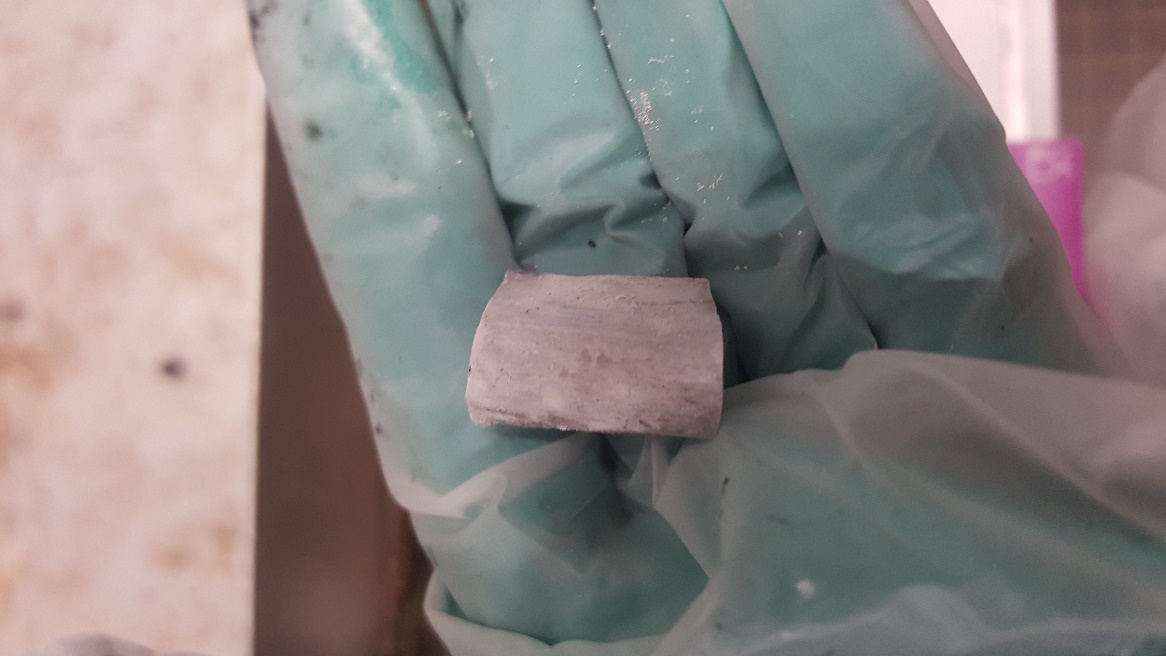 |
| --- | --- | --- | --- | --- | --- |
| 04018R-Lt * | 2.3 | 670 | 40 | Almost fully white with slight tinge of light grey, sawn edges are white, spongy layer shows separation from cortical bone, no HIF | 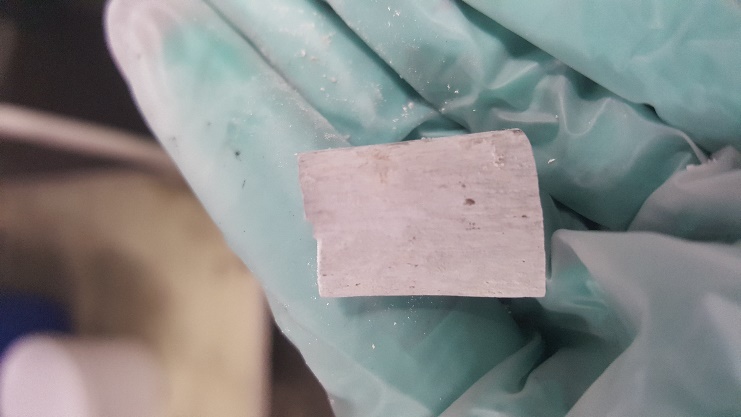 |
| 04018R-Lt | 2.1 | 690 | 25 | Largely grey/grey-blue with patches of white, slight cracking of bone seen | 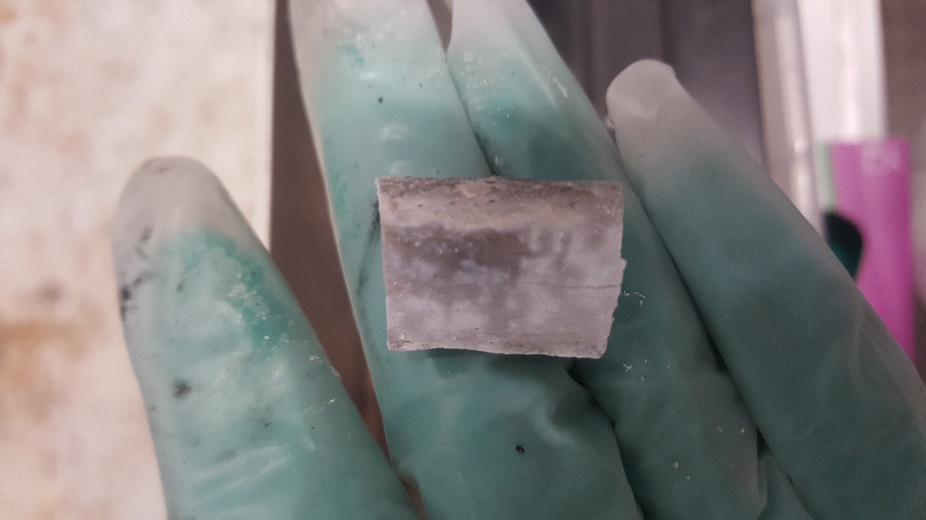 |
| 04018R-Lt * | Epiphysis 5.6 | 690 | 35 | 95% white with some grey areas and spots of yellowish-orange; longitudinal and curved transverse HIFs seen | 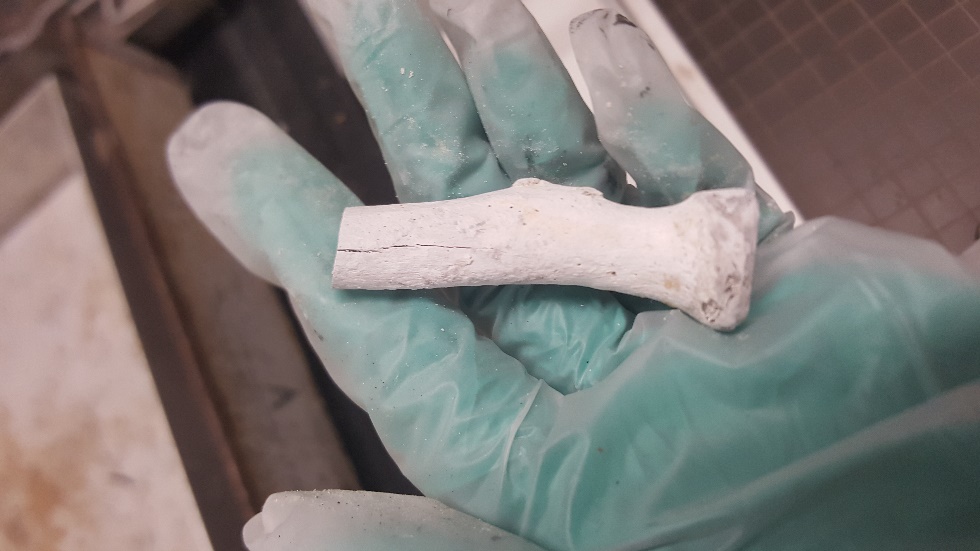 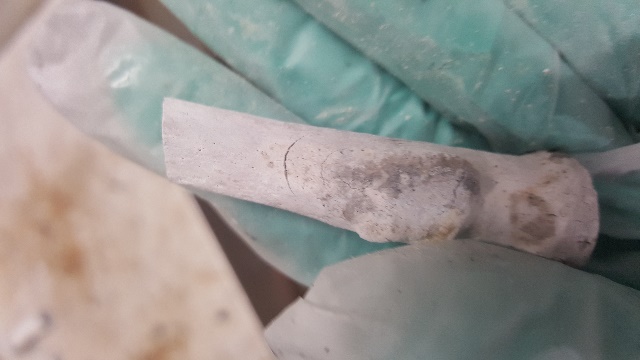 |
| 10817R-Rt * | 4.1 | 680 | 30 | Mostly white with tinge of grey, sawn edge is white, no HIF | 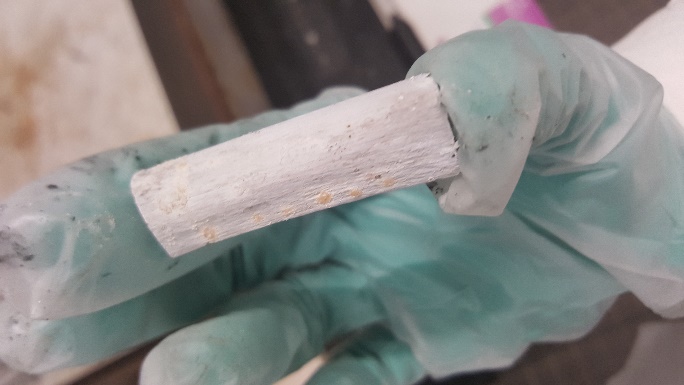 |

***Table S7 2/2****: Observations from the pilot study to identify the ideal temperature and duration of burning for the inversion to calcination stage to occur. Asterisk denotes the aforementioned factors apt for the desired stage of burning.*

# ESM section 4 – Grouping of bones for main study and distribution of bones across the 3 groups

| **Group Name** | **Original specimen number** | **New specimen label** |  |
| --- | --- | --- | --- |
| *Control-Burn (*A) | 12117R-Lt | RA1 |  |
|  | 12117U-Rt | UA2 |  |
|  | 08017R-Rt | RA3 |  |
|  | 06217U-Rt | UA4 |  |
|  | 09317R-Lt | RA5 |  |
|  | 08017U-Lt | UA6 |  |
|  | 18017R-Lt | RA7 |  |
|  | 18017U-Rt | UA8 |  |
|  | 02117R-Rt | RA9 |  |
|  | 10817U-Rt | UA10 |  |
| *Control-BFI (B)* | 1313R-Lt | RB1 |  |
|  | 12117U-Lt | UB2 |  |
|  | 06217R-Rt | RB3 |  |
|  | 08017U-Rt | UB4 |  |
|  | 09317U-Lt | UB5 |  |
|  | 08017R-Lt | RB6 |  |
|  | 09317U-Rt | UB7 |  |
|  | 18017U-Lt | UB8 |  |
|  | 02117U-Rt | UB9 |  |
|  | 02117R-Lt | RB10 |  |
| *BFI & Burn (C)* | 1313U-Lt | UC1 |  |
|  | 12117R-Rt | RC2 |  |
|  | 06217R-Lt | RC3 |  |
|  | 06217U-Lt | UC4 |  |
|  | 09317R-Rt | RC5 | |
|  | 10817U-Lt | UC6 | |
|  | 5313R-Lt | RC7 | |
|  | 5313U-Lt | UC8 | |
|  | 18017R-Rt | RC9 | |
|  | 02117U-Lt | UC10 | |

***Table S8****: The new labels for each bone specimen, after the 30 bones were separated into 3 groups. Abbreviation for specimen labels: R- radius, U- ulna, A/B/C- group name, X- number of bones in that group (1 to 10).*


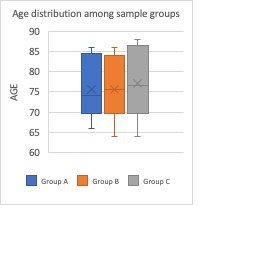

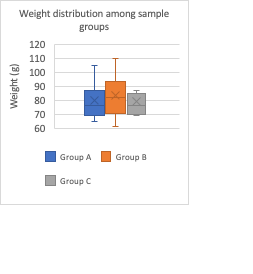

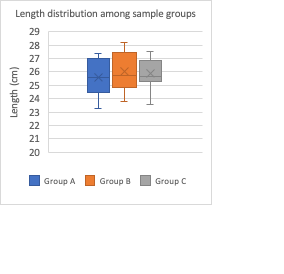


***Fig. S9****: Box plot showing the distribution of bone samples in each study group, based on age (****left****), weight (****center****) and length (****right****) of respective cadavers. Each group mostly has bones from adults ranging between 70-84 years old. Each group mostly has bones weighing between 70-85g. Groups A and B each contain one of the 2 heaviest bones. Every group mostly has bones ranging between 25-27cm. Each group also contains one of the 3 shortest bones.*

| **Sex** | **Group A** | **Group B** | **Group C** |  | **Number of radius and ulna** | **Group A** | **Group B** | **Group C** |
| --- | --- | --- | --- | --- | --- | --- | --- | --- |
| **Male** | 5 | 5 | 6 |  | **Radius** | 5 | 4 | 5 |
| **Female** | 5 | 5 | 4 |  | **Ulna** | 5 | 6 | 5 |

**Table S10**: Indicates the distribution of bones from males and females (**right**) and radius/ulna (**left**) in every group. Due to inequality in the total number of bones from males (16) and total number of bones from females (14) for the main study, Group C has more bones from males than females. Due to inequality in the total number of radii (14) and total number of ulna (16) for the main study, Group B has more ulna than radii.

# ESM section 5 – Scoring of each fracture feature

| **Feature** | | **Corresponding numerical label/score** |
| --- | --- | --- |
| **Fracture category** | Complete-simple | 1 |
|  | Complete-comminuted | 2 |
|  | Incomplete | 3 |
| **Fracture outline** | Helical/ curved | 1 |
|  | Transverse | 2 |
|  | Longitudinal and transverse | 3 |
|  | Diagonal | 4 |
|  | Diagonal with a step | 5 |
|  | Columnar | 6 |
| **Fracture location** | Proximal | 1 |
|  | Intermediate | 2 |
|  | Distal | 3 |
| **Fracture surface** | Smooth | 1 |
|  | Rough | 2 |
|  | Rough and smooth | 3 |
| **Fracture type** | Transverse | 1 |
|  | Oblique | 2 |
|  | Spiral | 3 |
|  | Comminuted | 4 |
|  | Segmental | 5 |
|  | Longitudinal | 6 |
| **State of burning** | Unmodified | 1 |
|  | Carbonized - Early | 2 |
|  | Carbonized - Complete | 3 |
|  | Partially burnt | 4 |
|  | Calcined - Partial | 5 |
|  | Calcined - Complete | 6 |
|  | Complete | 7 |
| **Colour of bone** | Black | 1 |
|  | Dark grey | 2 |
|  | Light grey | 3 |
|  | Grey (balance of light & dark) | 4 |
|  | Blue-Grey | 5 |
|  | White | 6 |
|  | Brown | 7 |
| **Type of HIF** | Longitudinal | 1 |
|  | Straight transverse | 2 |
|  | Curved transverse | 3 |
|  | Step | 4 |
|  | Patina | 5 |
|  | Delamination | 6 |
|  | Warping | 7 |
| **Temperature distribution (from colourimetry)** | 300°C -600°C | 1 |
|  | 450°C -700°C | 2 |
|  | 700°C -900°C | 3 |
|  | > 900°C | 4 |

***Table S11****: Scoring labels for each fracture feature. The scoring was assigned randomly, not in any particular order. The colourimetric clusters shown here are simplified from those in the study by Krap et al. (2019, see bibliography manuscript) to correspond to the phase of inversion to calcination investigated in this study and for easier results analysis.*

# ESM section 6 – Images of bones before and after fracture production and/or burning

| **Specimen label** | **Pre-Burning** | **Post-Burning** |
| --- | --- | --- |
| **RA1** | 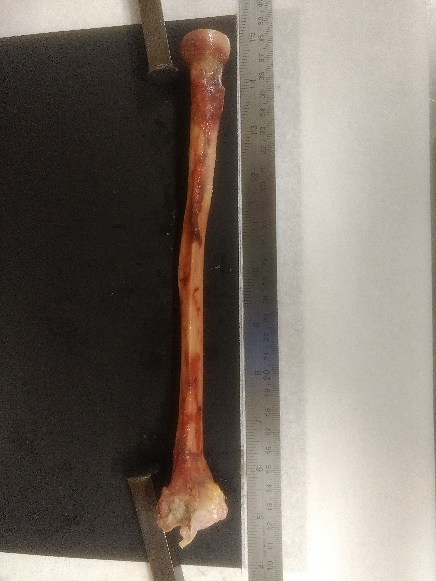 | 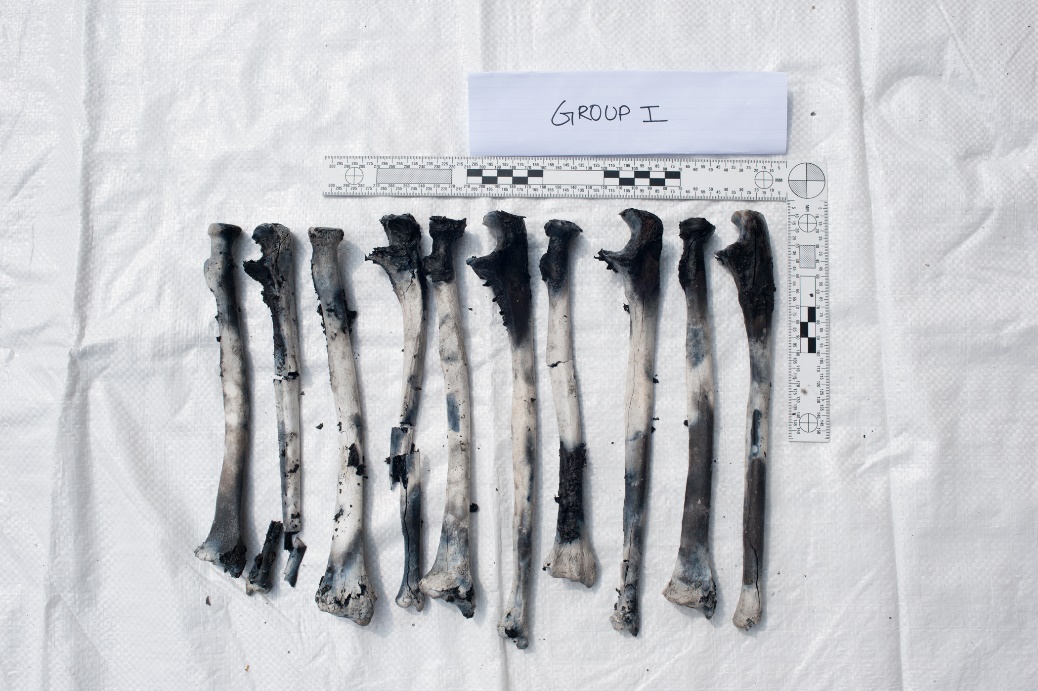  ******NOTE:*** *The burned bones shown are not in the same order as the unburned bones shown in this table.* |
| **UA2** | 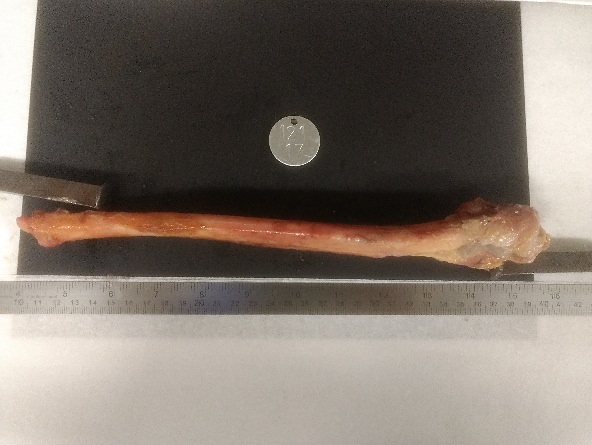 |  |
| **RA3** | 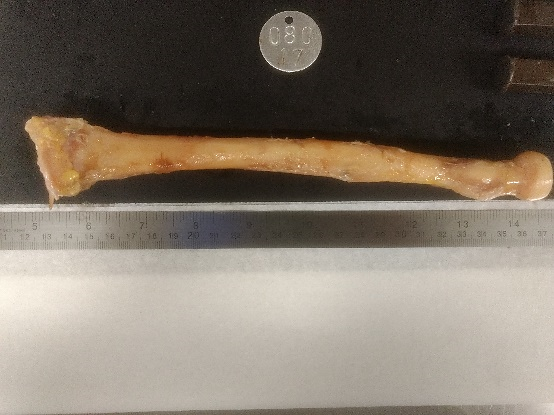 |  |
| **UA4** | 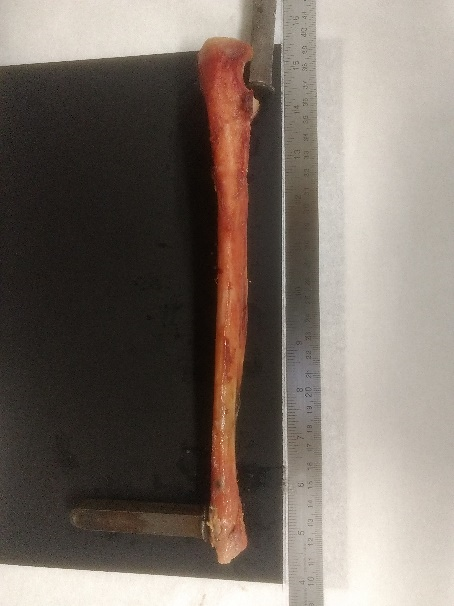 |  |
| **RA5** | 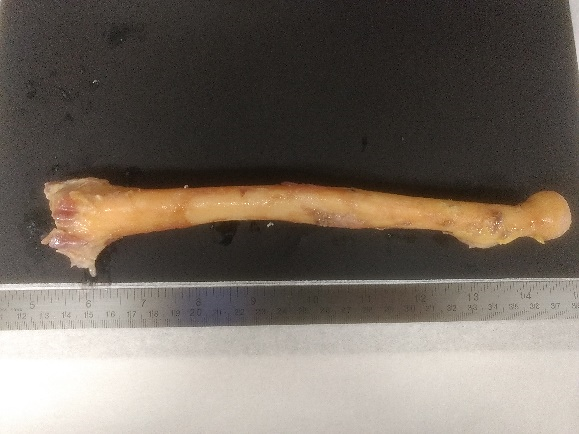 |  |
| **UA6** | 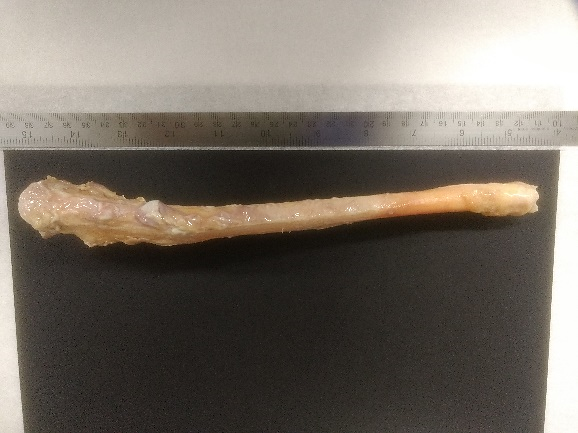 |  |
| **RA7** | 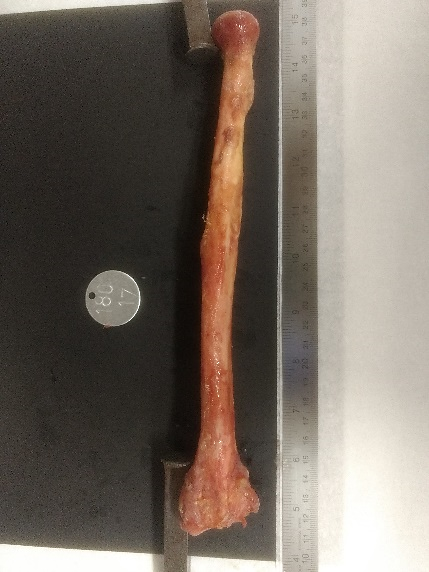 |  |
| **UA8** | 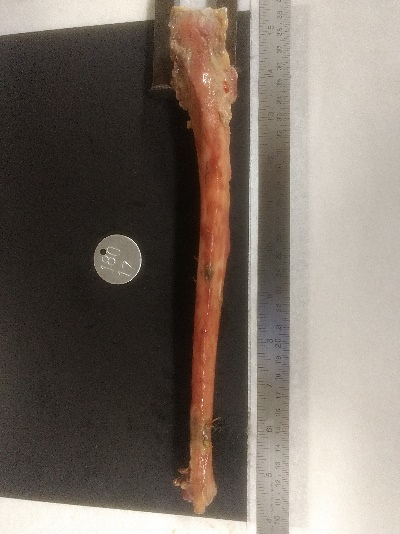 |  |
| **RA9** | 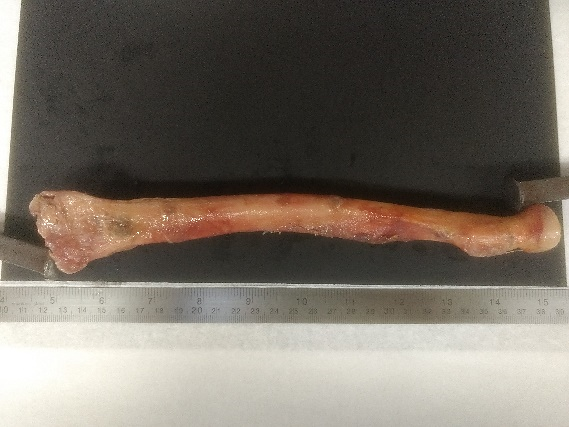 |  |
| **UA10** | 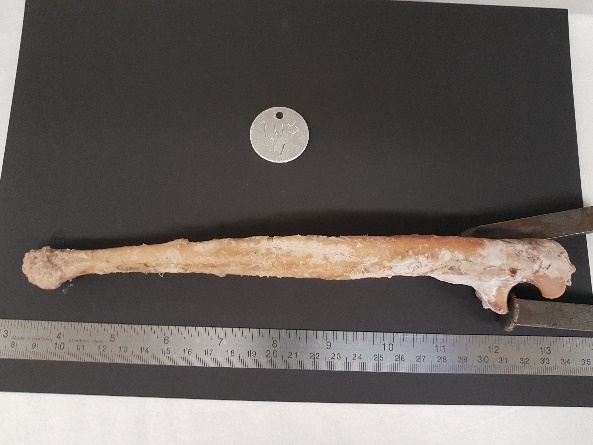 |  |

***Fig. S12****: Pictures of bones from Group A – before and after burning.*

| **Specimen label** | **Post-BFT** |
| --- | --- |
| **RB1** | 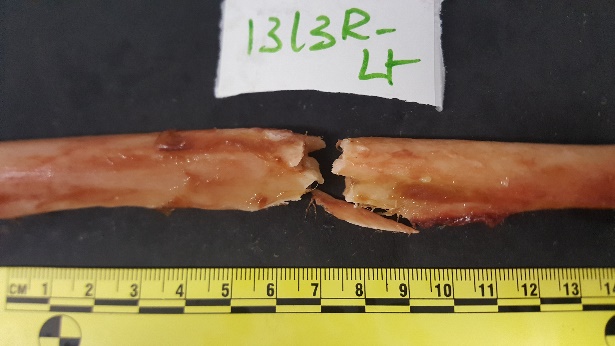 |
| **UB2** | 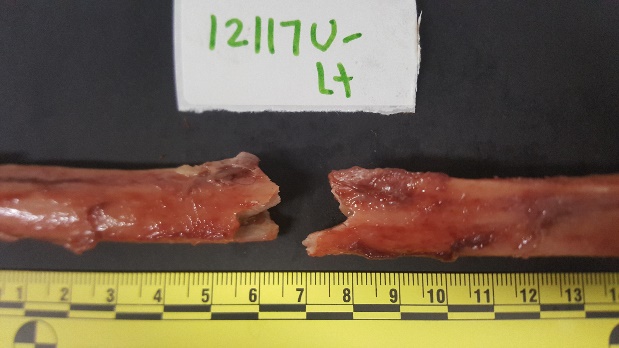 |
| **RB3** | 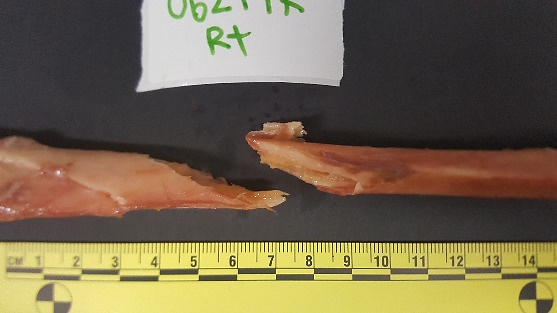 |
| **UB4** | 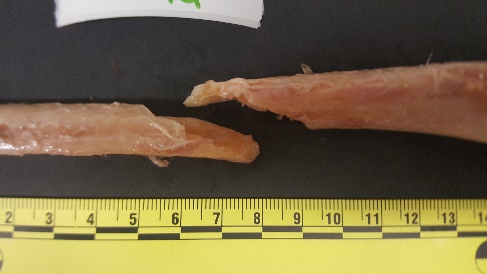 |
| **UB5** | 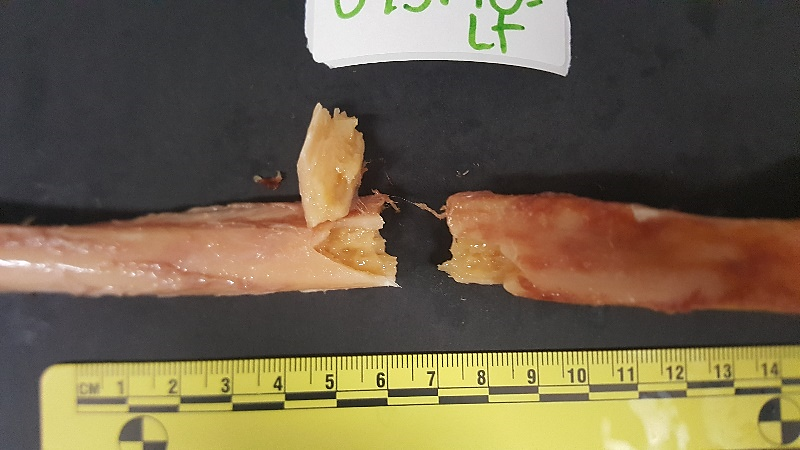 |
| **RB6** | 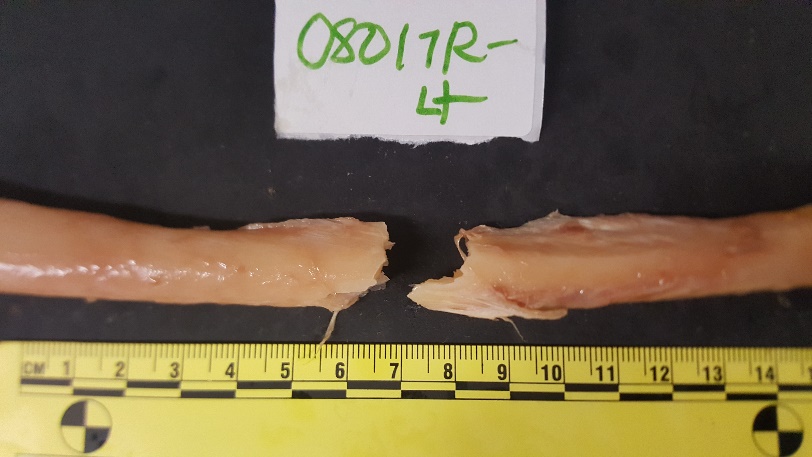 |
| **UB7** | 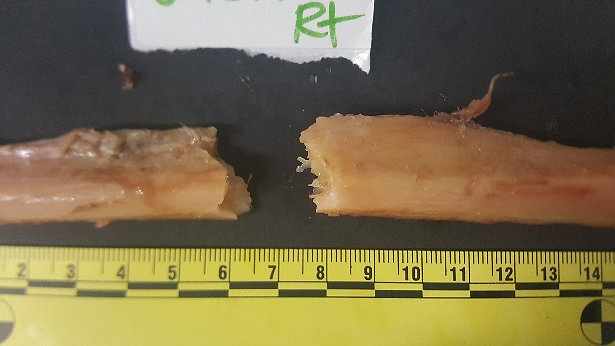 |
| **UB8** | 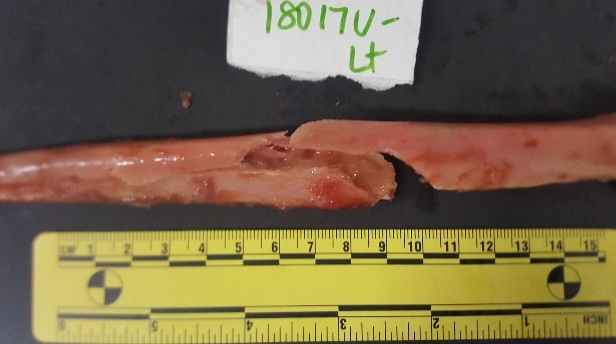 |
| **UB9** | 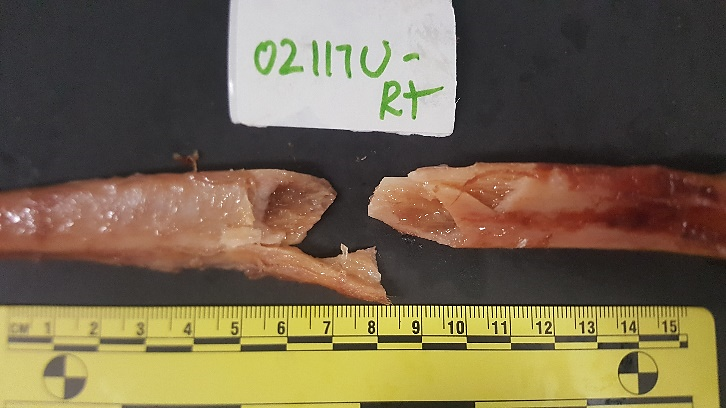 |
| **RB10** | 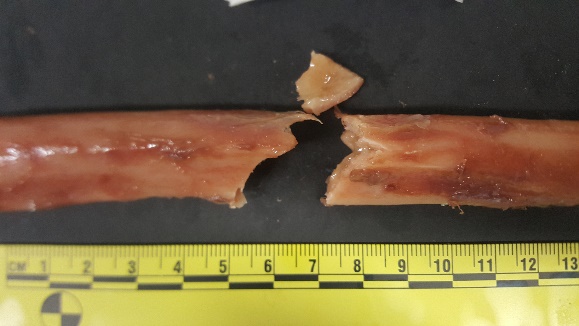 |

***Fig. S13****: Pictures of bones from Group B – after BFI.*

| **Specimen label** | **Pre-Burning** | **Post-Burning** |
| --- | --- | --- |
| **UC1** | 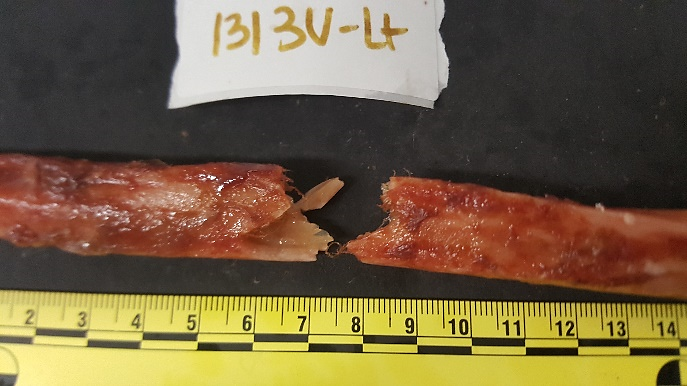 | 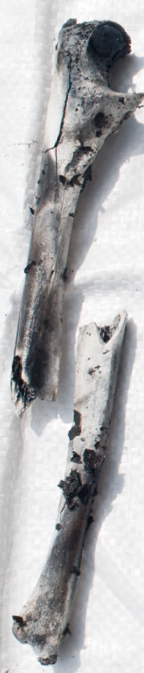 |
| **RC2** | 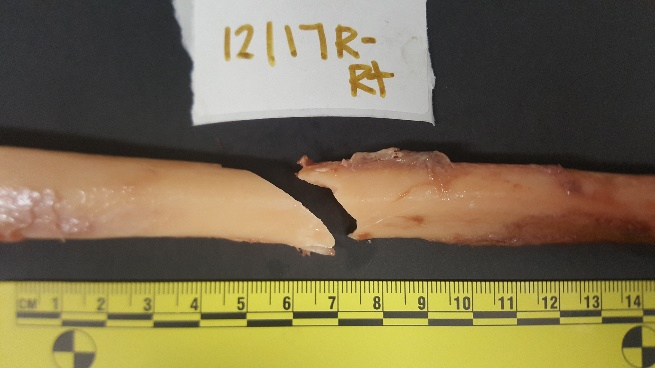 | 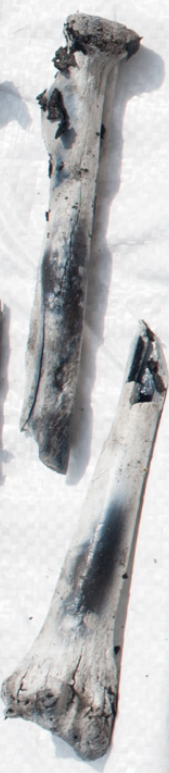 |
| **RC3** | 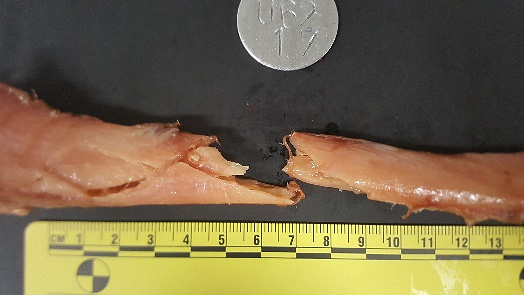 | 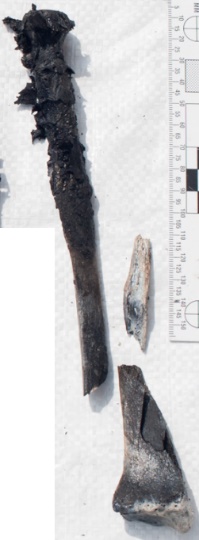 |
| **UC4** | 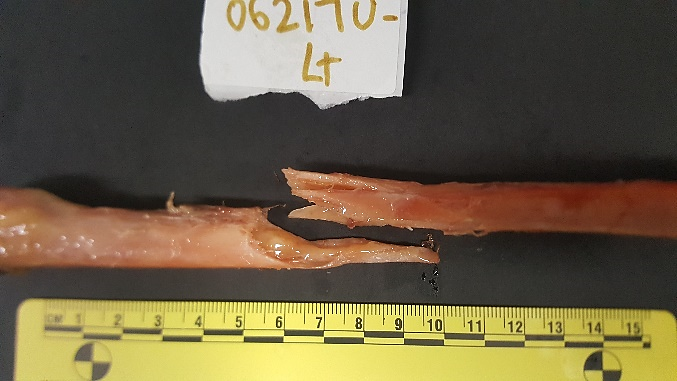 | 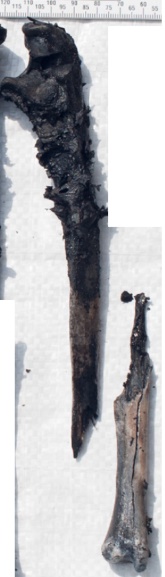 |
| **RC5** | 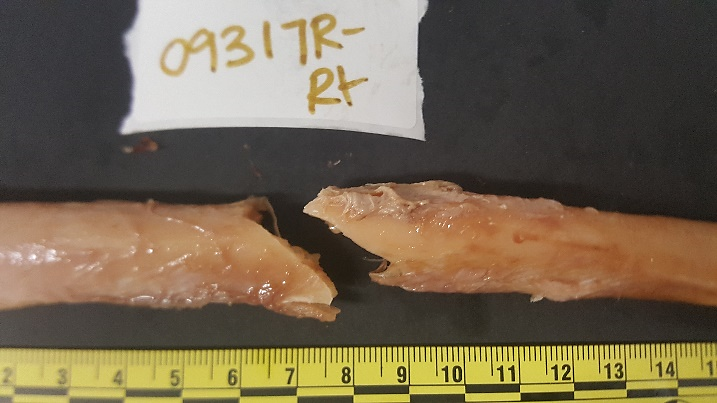 | 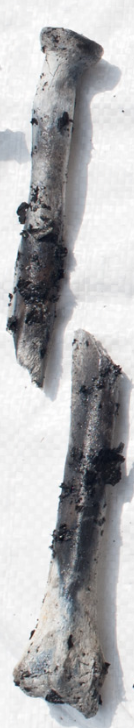 |
| **UC6** | 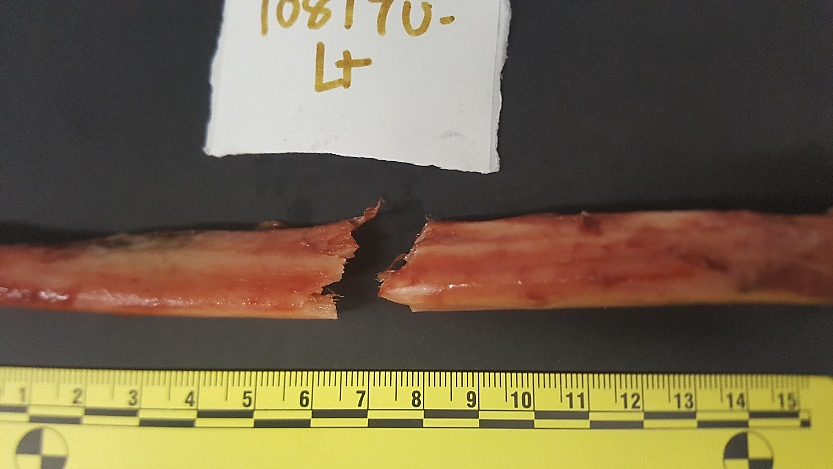 | 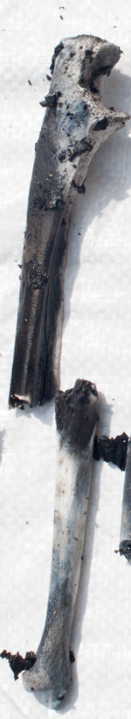 |
| **RC7** | 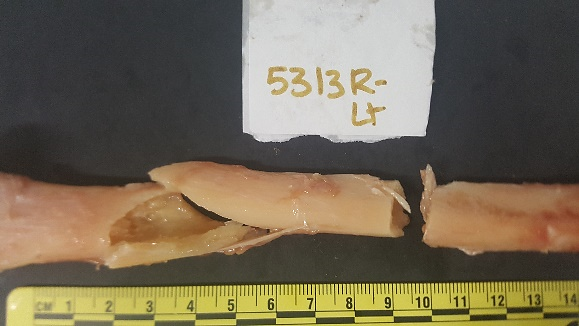 | 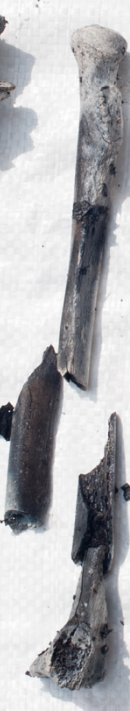 |
| **UC8** | 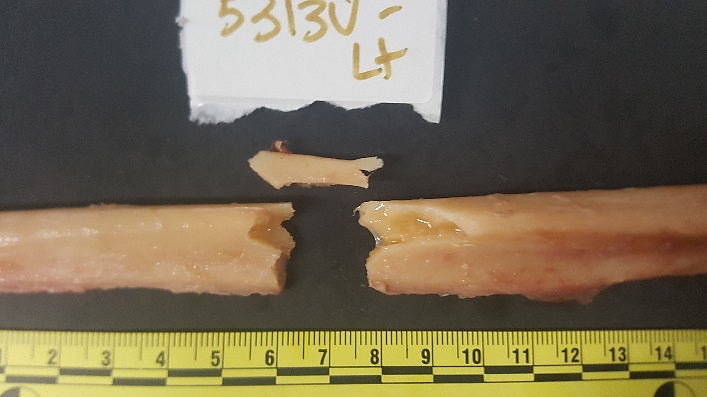 | 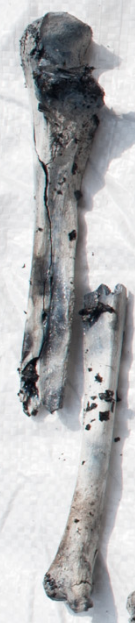 |

***Fig. S14 1/2****: Pictures of bones from Group C – after BFI (pre-burning) and after burning.*

| **RC9** | 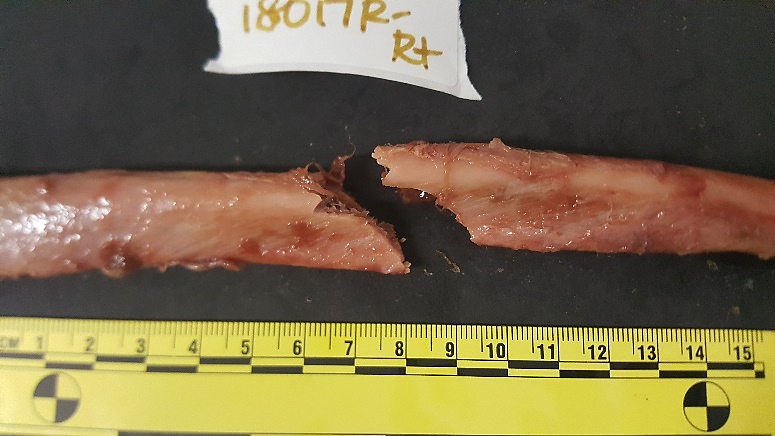 | 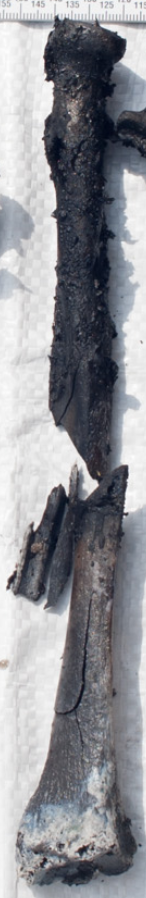 |
| --- | --- | --- |
| **UC10** | 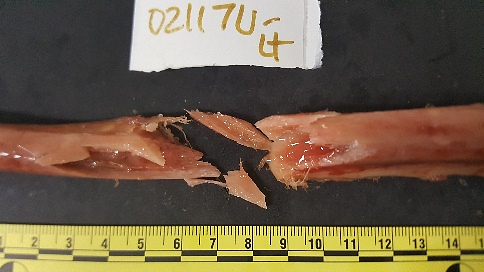 | 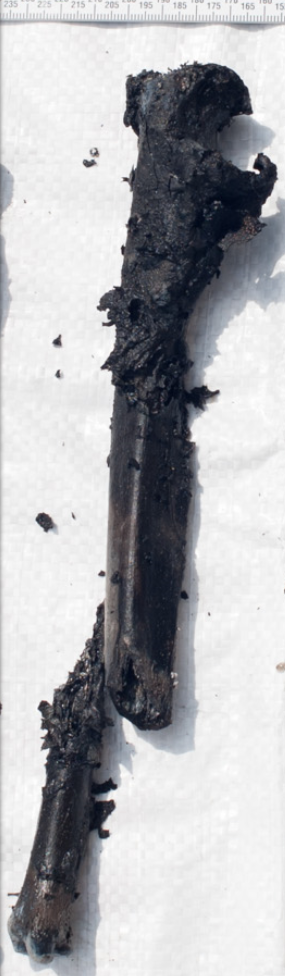 |

***Fig. S14 2/2****: Pictures of bones from Group C – after BFI (pre-burning) and after burning.*

# ESM section 7 – Results for temperature distribution from colourimetric analysis and corresponding number and type of fractures


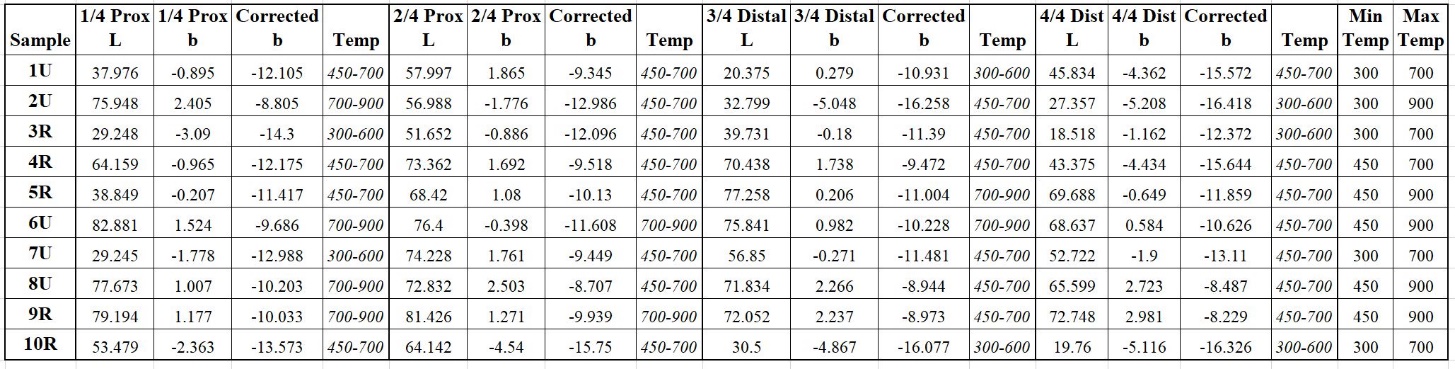
***Table S15****: Colourimetry results for Group A.*


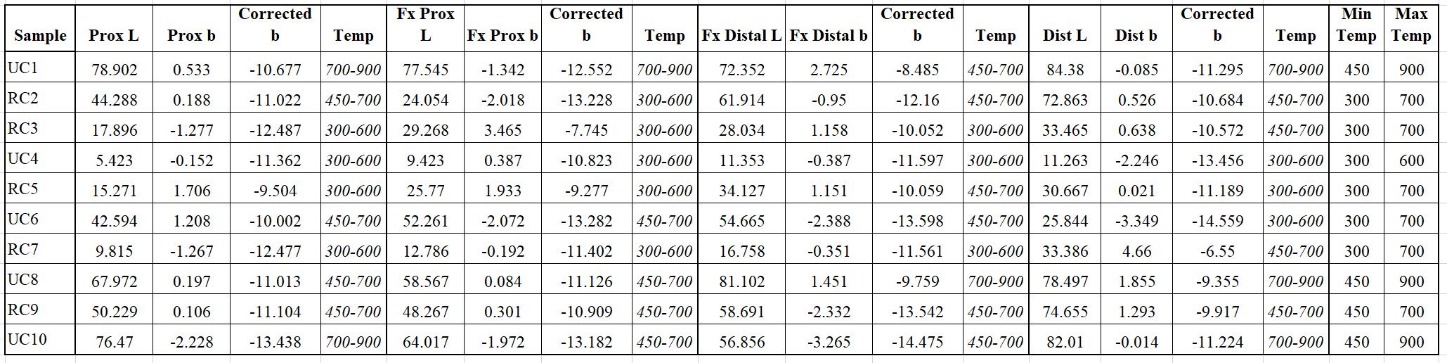


***Table S16****: Colourimetry results for Group C.*

| **Group A** | **Average Temperature Epiphyses** | **Average Temperature Intermediate** | **Fx Epiphyses** | **Fx Intermediate** | **Epiphyses HIBF** | **Intermediate HIBF** | **Epiphyses iHIBF** | **Intermediate iHIBF** |
| --- | --- | --- | --- | --- | --- | --- | --- | --- |
| **1U** | 450-700 | 450-700 | 2 | 4 | 2 | 2 | 0 | 2 |
| **2U** | 450-700 | 450-700 | 5 | 4 | 2 | 2 | 3 | 2 |
| **3R** | 300-600 | 450-700 | 2 | 2 | 1 | 2 | 1 | 0 |
| **4R** | 450-700 | 450-700 | 2 | 2 | 1 | 2 | 1 | 0 |
| **5R** | 450-700 | 700-900 | 1 | 4 | 0 | 2 | 1 | 2 |
| **6U** | 450-700 | 700-900 | 3 | 3 | 2 | 0 | 1 | 3 |
| **7U** | 450-750 | 450-700 | 2 | 3 | 0 | 1 | 2 | 2 |
| **8U** | 450-700 | 450-700 | 2 | 3 | 2 | 2 | 0 | 1 |
| **9R** | 450-700 | 700-900 | 0 | 3 | 0 | 2 | 0 | 1 |
| **10R** | 450-700 | 450-700 | 2 | 3 | 2 | 2 | 0 | 1 |
| ***Average***  ***Temperature/ No. of Fx*** | *450-700* | *450-700* | *21* | *31* | *12* | *17* | *9* | *14* |

***Table S17****: Table shows the average temperature at the epiphyseal and intermediate regions for each bone in Group A. The total number of fractures at each of these two regions as well as the number of each fracture type (HIBFs and iHIBFs) at these two regions are also tabulated.*

| **Grp C** | **Average Temperature Epiphyses** | **Average Temperature Intermediate** | **Fx Epiphyses** | **Fx Intermediate** | **Epiphyses HIBF** | **Intermediate HIBF** | **Epiphyses BFI** | **Intermediate BFI** | **Epiphyses iHIBF** | **Intermediate iHIBF** |
| --- | --- | --- | --- | --- | --- | --- | --- | --- | --- | --- |
| **UC1** | 450-700 | 450-700 | 3 | 5 | 2 | 2 | 0 | 2 | 1 | 1 |
| **RC2** | 700-900 | 450-700 | 5 | 5 | 2 | 1 | 0 | 2 | 3 | 2 |
| **RC3** | 300-600 | 450-700 | 3 | 3 | 0 | 0 | 1 | 1 | 2 | 2 |
| **UC4** | 450-700 | 300-600 | 2 | 2 | 1 | 1 | 1 | 1 | 0 | 0 |
| **RC5** | 450-700 | 700-900 | 2 | 4 | 2 | 1 | 0 | 2 | 0 | 1 |
| **UC6** | 450-700 | 450-700 | 2 | 4 | 2 | 2 | 0 | 2 | 0 | 0 |
| **RC7** | 450-700 | 450-700 | 2 | 3 | 0 | 0 | 0 | 2 | 2 | 1 |
| **UC8** | 700-900 | 450-700 | 3 | 4 | 2 | 1 | 0 | 2 | 1 | 1 |
| **RC9** | 300-600 | 300-600 | 2 | 4 | 0 | 0 | 0 | 2 | 2 | 2 |
| **UC10** | 300-600 | 300-600 | 2 | 2 | 1 | 1 | 1 | 1 | 0 | 0 |
| ***Average***  ***Temperature/***  ***No. of Fx*** | *450-700* | *450-700* | *26* | *36* | *12* | *9* | *3* | *17* | *11* | *10* |

***Table S18****: Table shows the average temperature at the epiphyseal and intermediate regions for each bone in Group C. The total number of fractures at each of these two regions as well as the number of each fracture type (HIBFs, BFIfx and iHIBFs) at these two regions are also tabulated.*

|  |  |
| --- | --- |
|  | |

**Fig. S19**: Bar graphs to illustrate the distribution of average burning temperature and number of fractures at epiphyses and intermediate regions of Group A bones. **a:** Shows the average burning temperature at the two regions for each bone, where 1 = 300°C-600°C, 2 = 450°C-700°C and 3 = 700°C-900°C. **b:** Shows the total number of fractures observed at the two regions for each bone. **c:** Shows the number of each fracture type (heat-induced, situational) observed at the two regions for each bone.

|  |  |
| --- | --- |
|  | |

**Fig. S20**: Bar graphs to illustrate the distribution of average burning temperature and number of fractures at epiphyses and intermediate regions of Group C bones. **a:** Shows the average burning temperature at the two regions for each bone, where 1 = 300°C-600°C, 2 = 450°C-700°C and 3 = 700°C-900°C. **b:** Shows the total number of fractures observed at the two regions for each bone. **c:** Shows the number of each fracture type (heat-induced, mechanically induced and situational) observed at the two regions for each bone.

# ESM section 8 – Dimensional changes

Since it was difficult to identify individual bones, to be able to make a before and after comparison, the bones in group Control-Burn (A) were evaluated as one group. There was an average of 1.13% shrinkage.

The bones in Group BFI & Burn (C) showed a maximum shrinkage of 4.61% and minimum of 0.75% for the proximal length (Mean: 2.63%) and maximum shrinkage of 19.31% and minimum of 1.64% for distal length (Mean: 8.44%). Some of the bones (5 out of 20) showed expansion of fracture length due to increased proximal and distal fracture lengths - maximum of 20.31% increase and minimum of 0.75% increase for fracture lengths.

# ESM section 9 – Measurement error

| **Sample number** | **Group A** | **Post-BFI Group B** | **Pre-burning Group C** | **Post-burning Group C** |
| --- | --- | --- | --- | --- |
| **1** | 78 ± 1.2 | 82 ± 1.8 | 29 ± 2.0 | 28 ± 1.2 |
| **2** | Nil | 64 ± 2.2 | 54 ± 1.9 | 57 ± 1.2 |
| **3** | Nil | 36 ± 3.4 | 59 ± 1.5 | 61 ± 1.2 |
| **4** | 78 ± 0.9 | 51 ± 2.1 | 23 ± 1.8 | 24 ± 0.9 |
| **5** | 67 ± 1.2 | 58 ± 2.3 | 41 ± 2.5 | 37 ± 1.5 |
| **6** | 87 ± 1.2 | 55 ± 1.2 | 55 ± 2.1 | 57 ± 1.5 |
| **7** | Nil | 87 ± 2.7 | RC7 | RC7 |
| **8** | Nil | 76 ± 2.6 | 29 ± 1.2 | 34 ± 0.9 |
| **9** | 66 ± 1.5 | 86 ± 1.8 | 39 ± 0.9 | 42 ± 0.9 |
| **10** | Nil | 54 ± 1.2 | 58 ± 1.5 | 56 ± 1.2 |
| **Average per group** | 1.2 | 2.13 | 1.711111111 | 1.166666667 |
| **Total average for all groups** | 1.551944445 | | | |

***Table S21****: Measurement error shown for each bone, each group and all groups combined.*

# ESM section 10 – Observations and measurements for each bone as per the features from the checklist

| **Specimen label** | **RB1** | **UB2** | **RB3** | **UB4** | **UB5** | **RB6** | **UB7** | **UB8** | **UB9** | **RB10** |
| --- | --- | --- | --- | --- | --- | --- | --- | --- | --- | --- |
| **No. of Fx** | 1 | 1 | 2 | 1 | 1 | 1 | 1 | 1 | 1 | 1 |
| **Fragmentation** | 1 | None | 1 (still attached) | None | 1 | None | None | 1 | 1 (still attached) | 1 (still attached) |
| **Fragmentation no. and size(s)** | Small -2.6cm | None | Small -1.2cm | None | Small -2.7cm | None | None | Medium -4.8cm | Medium -3.4cm | Small -1.6cm |
| **Fx category** | Complete- comminuted | Complete-simple | Complete-comminuted & Incomplete | Complete-simple | Complete- comminuted | Complete-simple | Complete-simple | Complete- comminuted | Complete- comminuted | Complete- comminuted |
| **Location on bone** | Intermediate | Intermediate | Distal | Intermediate | Intermediate | Intermediate | Intermediate | Intermediate | Intermediate | Intermediate |
| **Fx outline** | Transverse with jagged edges; sharp; clearly defined | Transverse with step in the middle; sharp; clearly defined | Helical/ curved & diagonal-incomplete; sharp shard with blunt curve; clearly defined | Helical/ curved & diagonal; sharp shard with blunt curve; clearly defined | Transverse with some jagged edges; sharp; clearly defined | Transverse with step; sharp; clearly defined | Transverse; sharp with some blunt edges; clearly defined | Longitudinal and transverse; sharp; clearly defined | Transverse & Helical/ curved (V-shaped); mostly blunt with one sharp region; clearly defined | Diagonal with a step & some jagged edges; sharp with some blunt edges; clearly defined |
| **Fx surface** | Smooth | Smooth | Smooth | Smooth | Smooth | Smooth | Smooth | Smooth | Smooth | Smooth |
| **Length of Fx (Proximal and Distal ends) (cm)** | Proximal: 11.5 Distal: 13.3 | Proximal: 14.3 Distal: 13.7 | Proximal: 14.8 Distal: 11.8 | Proximal: 13.7 Distal: 14.8 | Proximal: 12.6 Distal: 11.8 | Proximal: 11.9 Distal: 11.8 | Proximal: 14.6 Distal: 12.2 | Proximal: 14.3 Distal: 17.2 | Proximal: 15.5 Distal: 14.6 | Proximal: 13.7 Distal: 12.6 |
| **Ratio of length of fracture on tension side to compression side (cm)** | Proximal: 11.5/11.2=1.03 Distal: 12.8/13.3=0.96 | Proximal: 13/14.3=0.91 Distal: 13.7/12.5=1.10 | Proximal: 12.1/14.8=0.82 Distal: 11.8/8.5=1.39 | Proximal: 13.7/10.4=1.32 Distal: 11.5/14.8=0.78 | Proximal: 12.6/11.6=1.09 Distal: 10.3/11.8=0.87 | Proximal: 11.9/11.5=1.03 Distal: 11.5/11.8=0.97 | Proximal: 14.6/14.4=1.01 Distal: 11.7/12.2=0.96 | Proximal: 14.3/9.9=1.44 Distal: 8.6/17.2=0.5 | Proximal: 15.5/13.2=1.17 Distal: 14.6/12.3=1.19 | Proximal: 13.7/12.9=1.09 Distal: 11.4/12.6=0.90 |
| **Fx angle (°)** | 82 | 64 | 36 | 51 | 58 | 55 | 87 | 76 | 86 | 54 |
| **Fx type** | Oblique | Oblique | Oblique | Spiral | Oblique | Oblique | Transverse | Oblique | Transverse | Oblique |

***Table S22****: Denotes the different observations for each feature for Group B (BFI only). The fracture angle is a mean of triplicate measurements.* ***Legend:*** *Fx -fracture, no. -number.* ***Note:*** *Angles shown in table are rounded to nearest whole number but for statistical analysis, the exact number was used.*

| **Specimen label** | **UC1** | **RC2** | **RC3** | **UC4** | **RC5** | **UC6** | **RC7 *** | **UC8** | **RC9** | **UC10** |
| --- | --- | --- | --- | --- | --- | --- | --- | --- | --- | --- |
| **No. of Fx** | 1 | 2 | 1 | 1 | 1 | 1 | 2 | 1 | 1 | 1 |
| **Fragmentation** | 1 (still-attached) | Yes (seemingly big fragment(s) **missing**) | None | 3 (all still-attached) | None | None | 2 (all still-attached, 1 slightly) | 1 | None | 5 (2 still attached) |
| **Fragmentation no. and size(s)** | Small -1cm | Unknown | None | Small -2.5cm & Medium -3.2cm,4.3cm | None | None | Medium -3.6cm, Large -6.9cm | Small -2.6cm | None | Small -1.3cm, 1.3cm, 1.5cm, 2.2cm, 2.7cm |
| **Fx category** | Complete-comminuted | Complete-comminuted | Complete-simple | Complete-comminuted | Complete-simple | Complete-simple | Complete-comminuted | Complete-comminuted | Complete-simple | Complete-comminuted |
| **Location on bone** | Intermediate | Intermediate | Distal | Distal | Intermediate | Intermediate | Distal & Intermediate (Large fragment) | Intermediate | Intermediate | Distal |
| **Fx outline** | Diagonal with a step; sharp; clearly defined | Helical/curved & small step with jagged edges; mostly blunt with some sharp edges; clearly defined | Helical/curved & longitudinal; sharp; clearly defined | Helical/curved; sharp; clearly defined | Helical/curved; sharp and blunt; clearly defined | Columnar/ Jagged; sharp; clearly defined | Helical/curved -Distal & Transverse/ Jagged -Intermediate; sharp -distal & blunt -intermediate; clearly defined | Diagonal & oblique at 130°; sharp; clearly defined | Helical/curved with jagged edges; sharp; clearly defined | Diagonal with a step; sharp; most regions are clearly defined |
| **Fx surface** | Smooth | Smooth | Smooth | Smooth | Smooth | Smooth | Smooth | Smooth | Smooth | Smooth |
| **Length of Fx (Proximal and Distal ends) (cm)** | Proximal:15.2 Distal:13.2 | Proximal: 13.4 Distal: 13.2 | Proximal: 17.6 Distal: 10.4 | Proximal: 19.1 Distal: 12.8 | Proximal: 13.2 Distal: 12.3 | Proximal: 13.5 Distal: 11.7 | Proximal: 12.9 Distal: 9.4 | Proximal: 12.2 Distal: 14.1 | Proximal: 12.9 Distal: 14.5 | Proximal: 6.4 Distal: 18.4 |
| **Ratio of length of fracture on tension side to compression side (cm)** | Proximal: 13.5/15.2=0.89 Distal: 11.5/13.2=0.87 | Proximal: 12/13.4=0.90 Distal: 13.2/10.3=1.28 | Proximal: 17.6/15.1=1.17 Distal: 7.5/10.4=0.72 | Proximal: 19.1/14.3=1.34 Distal: 8/12.8=0.63 | Proximal: 11.3/13.2=0.86 Distal: 12.3/10.3=1.19 | Proximal: 13.5/13=1.04 Distal: 11.7/10.9=1.07 | Proximal: 12.9/12.8=1.01  Distal: 5.5/9.4=0.59 | Proximal: 12.2/12=1.02 Distal: 14.1/11.8=1.19 | Proximal: 11/12.9=0.85 Distal: 14.5/12.5= 1.16 | Proximal: 6.4/4=1.6 Distal: 13.8/18.4=0.75 |
| **Fx angle (°)** | 29 | 54 | 59 | 23 | 41 | 55 | 87 | 29 | 39 | 58 |
| **Fx type** | Oblique | Oblique | Spiral | Comminuted | Spiral | Oblique | Segmental | Oblique | Oblique | Comminuted |

***Table S23****: Denotes the different observations for each feature for Group C (BFI & Burning), before the burning experiment. The fracture angle is a mean of triplicate measurements.* ***Legend:*** *Fx -fracture, no. -number.* ***Note:*** *Angles shown in table are rounded to nearest whole number but for statistical analysis, the exact number was used. * RC7 was not statistically analysed due to foreseeable large deviation in data for fracture type.*

| **Specimen label** | **1U** | **2U** | **3R** | **4R** | **5R** | **6U** | **7U** | **8U** | **9R** | **10R** |
| --- | --- | --- | --- | --- | --- | --- | --- | --- | --- | --- |
| **State of burning** | Partial calcination | Partial calcination | Partially burnt | Partial calcination | Partial calcination | Partial calcination | Partial calcination | Partial calcination | Partial calcination | Partial calcination |
| **# Fracture** | None | 1 | None | None | 1 | 1 | 2 | None | None | 1 |
| **Fragmentation #** | None | 1 | None | None | None | None | None | None | None | None |
| **Fragmentation Size(s)** | None | Medium -3.6cm | None | None | None | None | None | None | None | None |
| **Colour Pattern** | Predominantly black towards epiphyseal parts with large regions of intermediate part being grey; all regions exhibit blue-grey tinge | Proximal epiphysis black, followed by light grey and patches of white, then a band of black around the bone and finally distal epiphysis being grey | Posterior bone is largely black; anterior distal epiphysis is black too; rest of the anterior bone is light and dark grey with blue tinges | Anterior bone is mostly black and dark grey with blue tinge and intermediate part (closer to proximal) is white; posterior bone is predominantly light grey and white with light blue tinges and black regions near the distal epiphysis and proximal epiphysis fully | Posterior mostly white and light grey with blue tinges; anterior is largely black with small region of white near the intermediate part of the bone | Predominantly white and light grey across the bone; small patch of black in middle and either epiphyses | Anterior mostly black and dark grey with light grey and white portion in the middle of diaphysis; posterior is predominantly light grey and white while distal epiphysis is black | Proximal epiphysis is black while remaining bone is predominantly white and light grey; some portions of black and blue-grey towards intermediate part of bone and distal epiphysis | Predominantly white and light grey; radial head, radial tuberosity and anterior distal epiphysis show black and dark grey regions with blue tinge | Posterior bone shows white at intermediate part of bone and black and dark grey with blue tinge on either epiphysis; anterior bone is predominantly dark-grey with some blue tinge and some light-grey regions |
| **Colour (Top 3)** | Black, grey, blue-grey | Black, Grey, White | Black, Grey, Blue-grey | Black, Grey, White | White, Black, Blue-grey | White, Light grey, Black | Black, Grey, White | White, Light grey, Black | White, Light grey, Black | Dark grey, Black, White |
| **Fracture Category** | None | Complete-comminuted | None | None | Complete-simple | Complete-simple | Complete-simple | None | None | Complete-simple |
| **Location on bone** | None | Distal | None | None | Intermediate | Intermediate | Proximal | None | None | Proximal |
| **Fracture outline** | None | Transverse with a step; blunt | None | None | Transverse with jagged edges; blunt | Transverse; blunt | Transverse; blunt | None | None | Longitudinal-transverse; blunt |
| **Fracture surface** | Smooth | Smooth | Smooth | Smooth | Smooth | Smooth | Smooth | Smooth | Smooth | Smooth |
| **Length of bone (cm)** | 26.5 | 26.3 | 23.1 | 25.2 | 25.3 | 25.3 | 24.7 | 27.7 | 25.5 | 23.1 |
| **HIF from fire** | Longitudinal HIF from epiphysis anterior proximal to epiphysis anterior distal; Straight transverse HIF at distal epiphysis; Curved transverse near proximal epiphysis; Clearly defined with HIF extending into bone/cavity | Longitudinal HIF from middle of diaphysis to posterior distal; Clearly defined with HIF extending into bone/cavity | Fine longitudinal HIFs along entire axis, both posterior and anterior; Clearly defined with HIF extending into bone/cavity | Longitudinal HIF from middle of diaphysis to anterior proximal; warping in middle of diaphysis posterior; Clearly defined with HIF extending into bone/cavity | Straight transverse HIF at intermediate portion of anterior bone; Clearly defined with HIF extending into bone/cavity | Warping seen, especially at epiphyses | Small longitudinal HIFs at posterior proximal epiphysis; Clearly defined with HIF extending into bone/cavity | Curved transverse anterior proximal; microstructural cracking at posterior proximal; longitudinal HIF posterior spanning diaphysis; Straight transverse HIF near posterior distal end; Clearly defined with HIF extending into bone/cavity | Longitudinal HIF posterior distal; cracking near BFT site posterior proximal & longitudinal HIF above this; warping posterior diaphysis; longitudinal HIF anterior distal; Clearly defined with HIF extending into bone/cavity | Straight transverse and longitudinal HIF posterior distal; longitudinal HIF anterior proximal end to distal end; Clearly defined with HIF extending into bone/cavity |
| **Situational Fx** | Longitudinal Fx at middle of diaphysis posterior; Clearly defined with Fx outline elevated on one side of Fx | Longitudinal Fx from epiphysis anterior proximal to epiphysis anterior distal; longitudinal-transverse Fx at distal end; Clearly defined with Fx outline elevated on one side of Fx | None | None | Longitudinal Fx from posterior distal to diaphysis, curving around anterior bone; transverse Fx in middle of diaphysis (broken along lines of straight transverse HIF); Clearly defined with Fx outline elevated on one side of Fx | Longitudinal Fx from anterior distal towards middle of diaphysis causing transverse Fx in the middle of diaphysis; Clearly defined with Fx outline elevated on one side of Fx | Transverse Fx near proximal end and transverse Fx near disal end; Clearly defined with Fx outline elevated on one side of Fx | None | None | Longitudinal-transverse Fx at roughly 1/3 of bone from proximal end; Clearly defined with Fx outline elevated on one side of Fx |
| **Fracture angle** | None | 78 | None | None | 78 | 67 | 87 | None | None | 66 |

***Table S24****: Denotes the different observations for each feature for Group A (only burning, no BFI). The fracture angle is a mean of triplicate measurements.* ***Legend:*** *Fx -fracture, no. -number.* ***Note:*** *Angles shown in table are rounded to nearest whole number but for statistical analysis, the exact number was used.*

| **Specimen label** | UC1 | RC2 | RC3 | UC4 | RC5 | UC6 | RC7 * | UC8 | RC9 | UC10 |
| --- | --- | --- | --- | --- | --- | --- | --- | --- | --- | --- |
| **State of burning** | Partial calcination | Partial calcination | Partially burnt | Partially burnt | Partial calcination | Partial calcination | Partial calcination | Partial calcination | Partially burnt | Complete carbonization |
| **# Fracture** | 1 | 1 | 1 | 1 | 1 | 1 | 2 | 1 | 2 | 1 |
| **Fragmentation #** | Yes | Yes | Yes | Yes | None | None | Yes | Yes | Yes | Yes |
| **Colour Pattern** | Posterior predominantly grey-white; anterior predominantly black-grey; at BFT site distal part is whiter while proximal part is black with a short white border (heat) | Mostly grey-white throughout; some black regions on the posterior; blue-grey tinge between these regions; BFT site dark grey; radial tuberosity dark grey | Proximal to middle of diaphysis is black; then becomes regions of brown (dark and light) and begins to become dark grey; proximal BFT site is black with small white border but becomes grey-white on distal side | Predominantly black; distal part is grey-brown; some brown regions especially near proximal part of BFT site | Anterior is predominantly light grey and patches of white; posterior shows almost equal spread of dark grey and light grey | Anterior proximal is predominantly black; BFT site shows brown burn and heat line; anterior distal is predominantly grey with spots of white; posterior proximal and distal are predominantly black and dark grey with blue tinge | Anterior proximal bone is mostly grey with spots of white and patch of dark grey with blue-grey tinge; anterior distal is black; posterior shows alternating bands of white-light grey and black, this pattern follows through the BFT site | Mostly light grey & white; intermediate BFT region shows dark grey and black with blue tinges spanning towards either epiphysis | Proximal bone is predominantly black; distal bone tends from black to dark grey and lighter grey at the epiphysis | Predominantly black throughout bone; beginning of dark-grey between posterior proximal and intermediate regions |
| **Colour (Top 3)** | Grey, Black, White | Grey,Black, Blue-Grey | Black, Grey, Brown | Black, Brown, Grey | Light grey, Dark grey, White | Black, Dark Grey, White | Black, Grey, White | Light Grey, White, Blue-grey | Black, Dark grey, Light grey | Black, Dark-grey |
| **Fracture Category** | Complete-comminuted | Complete-comminuted | Complete-comminuted | Complete-comminuted | Complete-simple | Complete-simple | Complete-comminuted | Complete-comminuted | Complete-comminuted | Complete-comminuted |
| **Location on bone** | Intermediate | Intermediate | Distal | Distal | Intermediate | Intermediate | Distal & Intermediate (Large fragment) | Intermediate | Intermediate | Distal |
| **Fracture outline** | Diagonal with a step; Mostly blunt; clearly defined | Helical/curved & small step; mostly blunt with some sharp edges; clearly defined | Helical/curved; Blunt; clearly defined | Helical/curved; Mostly blunt with one sharp shard; clearly defined | Helical/curved; Blunt; Clearly defined | Columnar; Mostly blunt with some sharp edges; Clearly defined | Helical/curved -Distal & Transverse/ Jagged -Intermediate; sharp -distal & blunt -intermediate; clearly defined | Diagonal; Blunt; Clearly defined | Diagonal; Blunt; Clearly defined | Diagonal with a step; Blunt; Clearly defined |
| **Fracture surface** | Rough | Rough with slanted/curved region still quite smooth | Rough with slanted/curved region still quite smooth | Rough with curved regions being smooth | Rough | Rough | Rough with curved regions being smooth | Mostly rough with some smoothness at slanted edges | Rough with slanted region quite smooth | Mostly rough with some regions beginning to roughen |
| **Length of Fx (proximal & distal) (cm)** | Proximal: 14.5 Distal: 12.9 | Proximal: 12.8 Distal: 12.1 | Proximal: 17.2 Distal: 8.7 | Proximal: 18.7 Distal: 11.7 | Proximal: 13.1 Distal: 13.5 | Proximal: 13.5 Distal: 11.4 | Proximal: 12.8 Distal: 9.1 | Proximal: 14.6 Distal: 12 | Proximal: 12.7 Distal: 11.7 | Proximal: 18.6 Distal: 7.7 |
| **Ratio of tension length to compression length** | Proximal: 13.9/14.5=0.96 Distal: 11.8/12.9=0.91 | Proximal: 12.8/11.9=1.08 Distal: 12.1/10.1=1.20 | Proximal: 15.3/17.2=0.90 Distal: 6.1/8.7=0.70 | Proximal: 18.7/17=1.1 Distal: 6.9/11.7=0.59 | Proximal: 11.1/13.1=0.85 Distal: 13.5/12.4=1.10 | Proximal: 13.4/13.5=0.99 Distal: 10.6/11.4=0.92 | Proximal: 12.8/10.8=1.19 Distal: 6.7/9.1=0.74 | Proximal:14.6/14.4=1.01 Distal: 11.6/12=0.97 | Proximal: 12.7/10.8=1.18 Distal: 11.7/10.7=1.09 | Proximal: 16.7/18.6=0.90 Distal: 6.7/7.7=0.87 |
| **HIF from fire** | Longitudinal HIF posterior proximal;cracking posterior proximal near BFT site and curved longitudinal HIF extending into BFT site and stopping there; Clearly defined with HIF extending into cavity/bone | Small long  itudinal HIF posterior proximal from epiphysis towards diaphysis; microstructural cracking at anterior proximal; step HIF near BFT (stops at BFT site) anterior distal; Clearly defined with HIF extending into cavity/bone | Heat border at posterior proximal side of BFT site | Brown burn posterior proximal at BFT site; longitudinal HIFs anterior distal; Clearly defined with HIF extending into cavity/bone | Longitudinal HIF extending from anterior distal to anterior proximal along BFT; Clearly defined with HIF extending into cavity/bone | Heat border at BFT site of proximal (around the bone); longitudinal HIF from epiphysis towards BFT site anterior distal; longitudinal from BFT site towards epiphysis anterior proximal; Clearly defined with HIFs extending into cavity/bone | Heat border near BFT and longitudinal-step HIFs along posterior proximal extending into epiphysis; Clearly defined with HIF extending into cavity/bone | Longitudinal HIF from BFT to epiphysis anterior distal; warping seen near anterior proximal end; Clearly defined with HIF extending into cavity/bone | None visible | Not so prominent longitudinal-step HIFs anterior proximal; Mostly clearly defined and fracture lines extending into cavity/bone |
| **Situational Fx** | Straight transverse meeting longitudinal HIF posterior distal; Clearly defined with Fx outline elevated on one side of longitudinal Fx | Longitudinal Fx in posterior proximal bone; Longitudinal Fx from posterior distal bone extending into middle of anterior diaphysis; Clearly defined with Fx outline elevated on one side of Fx | Longitudinal Fx extending from BFT anterior proximal; Clearly defined with Fx outline elevated on one side of Fx | None | Step-transverse Fx at anterior distal portion; Clearly defined with Fx outline elevated on one side of Fx | Curved longitudinal at BFT site posterior distal; Clearly defined with Fx outline elevated on one side of Fx | Longitudinal Fx from BFT anterior distal; Clearly defined with Fx outline elevated on one side of Fx | Longitudinal HIF from BFT to epiphysis anterior proximal; Clearly defined with Fx outline elevated on one side of Fx | Longitudinal HIF extending from anterior distal to anterior proximal; Clearly defined with Fx outline elevated on one side of Fx | None |
| **BFT Fx** | Oblique | Oblique | Comminuted | Comminuted | Spiral | Oblique | Segmental | Oblique | Comminuted | Comminuted |
| **Fracture angle** | 28 | 57 | 61 | 24 | 37 | 57 | 77 | 34 | 42 | 56 |

***Table S25****: Denotes the different observations for each feature for Group C (BFI & burning), after the burning experiment. The fracture angle is a mean of triplicate measurements.* ***Legend:*** *Fx -fracture, no. -number.* ***Note:*** *Angles shown in table are rounded to nearest whole number but for statistical analysis, the exact number was used. * RC7 was not used for statistical analysis.*

# ESM section 11- Glossary

| **GLOSSARY** | | |
| --- | --- | --- |
| **FEATURE** | | **DESCRIPTION** |
| Traumatic fractures* | Transverse fracture | Fracture occurs on the diaphysis at approximately right angles to the long axis of the bone, on at least 75% of the bone circumference. |
|  | Oblique fracture | Fractures run diagonally across the diaphysis, usually at about a  45-degree angle, on at least 75% of the bone circumference. |
|  | Spiral  fracture | Spiral fractures begin as small defects, then the cracks follow the peak of the tensile loading around the bone. A true spiral fracture involves a fracture line that traverses in two different oblique directions, on at least 75% of the bone circumference. |
|  | Comminuted fracture | More than two separate fragments are generated from fracture. |
|  | Segmental fracture | Multiple fractures leave diaphyseal portions separated from the proximal or the distal ends, the intervening segment is called a segmental fracture. It is a type of comminuted fracture with well-defined, large fragments are produced. |
| Heat-induced fractures | Longitudinal | Fracture follows the grain of the bone but could also follow a helical path down the bone’s long axis. |
|  | Straight transverse | Fracture is perpendicular to the longitudinal axis of the bone, on at least 75% of the bone circumference. The fracture may also penetrate through to the medullary cavity and might even cause complete transection. |
|  | Curved transverse | Fractures look like concentric circles or form coning, which the curvature of fracture tends to move towards one direction. They result from soft tissue pulling away bone surface during burning. |
|  | Step | Fracture spread from margin of longitudinal fractures, in a transverse manner, across diaphysis. Step fractures thus, frequently tend to be associated with longitudinal fractures. |
|  | Patina | Mesh of fine uniform pattern of cracks, resembling old painting cracks. These cracks are superficial to bone surface and do not enter the medullary cavity of bone. |
|  | Delamination | Splitting or flaking of bone layers (cortical from cancellous/spongy), appear as if top bone layer is peeling from the underlying one. |
| State of burning/ Colouration of bone fragments  (**Fig. S27**) | Unmodified | No visual features of thermal modification to bone; no colour change. |
|  | Carbonized | **Early carbonization:** (Dark-) brown-coloured regions of bone  **Complete carbonization:** Blackened bones (at-least 75% carbonization). |
|  | Partially burnt | More than 75% carbonization of bone with apparent grey/white regions |
|  | Calcined | **Partial calcination:** Grey and/or grey-blue coloured bone (50% or more, of bone) with some carbonized regions and/or white regions  **Complete calcination:** White-coloured bone (more than 75% of bone) with some grey regions |
|  | Complete | Bones become ashes (at-least 90% of full bone) |
|  | ***Note:*** | One bone may display combination of different states of burning, which will be accordingly noted in checklist |
| Fragmentation | | Breakage of bones into smaller pieces and corresponding number of fragments generated from impact on bone. A piece of bone being more than 1cm in any one dimension is considered one fragment.  The size of fragments will be denoted as small (<3 cm), medium (3–5 cm) or large (>5 cm). |

***Table S26 1/2****: Glossary for the used terminology including a description per feature.*

***: *Type of fracture for BFI needs to be determined based on consideration of other features, especially fracture category, outline and angle, collectively.***

| Fracture category | Complete-simple | Two separate and whole pieces of bone formed from fracture due to discontinuity in bone. |
| --- | --- | --- |
|  | Complete-comminuted | More than two separate pieces of bone formed from fracture – fragments are present. |
|  | Incomplete | Fractured pieces of bone still joined to main portion of bone (retention of bone continuity). |
| Fracture outline  (**Fig. S28**) | Helical/ curved | Fracture spirals its way round the bone shaft, at least 25% of the circumference. |
|  | Transverse | Fracture outline is perpendicular to longitudinal axis of bone, within 75° - 105° of the longitudinal axis. |
|  | Longitudinal and transverse | Fracture extends along the long axis pf the bone then becomes perpendicular (85° to 95°) to the same axis. |
|  | Diagonal | Fracture is oblique to longitudinal axis of bone, as a diagonal ‘cut’. |
|  | Diagonal with a step | Fracture begins as oblique to longitudinal axis of bone, then slightly penetrate into long axis of bone. Finally, the fracture continues as oblique ‘cut’. |
|  | Columnar | Uneven “steps” in an apparent diagonal manner, to the long axis of the bone. |
|  | Sharp | Fracture shards (extending bone) are visible and the fracture edges are sharp. Edge is considered sharp when a light dent forms upon pressing the edge softly against a worn glove. |
|  | Blunt | Fracture shards (extending bone) maybe visible (or not) and the fracture edges are blunt. |
|  | Clearly defined | Fracture edges are distinct, and the shape of outline can be identified. |
|  | Not clearly defined | Fracture edges are not distinct, and the shape of outline cannot be identified properly. |
| Fracture surface morphology | Smooth | Even and fine texture of fracture surface (cortical bone), covering at least 25% of that surface. |
|  | Rough | Uneven or bumpy/irregular texture of fracture surface (cortical bone), covering at least 25% of that surface. |
| Length of fracture | | Measured as longest/shortest shard of fracture on the tension side from the epiphyses (olecranon/radial head or styloid process) and the same on the compression side. Whichever length is longer, is taken as the length of fracture. Measurements taken from both proximal and distal ends of the bone. |
| Ratio of fracture length | | Taken as ratio of length from longest shard to epiphyses on tension side to the same on the compression side. |
| Fracture angle  (**Fig. S28**) | Right angle | Angle between fracture surface and the long axis of the bone is 90° (approximately 85°-95°) and this angle covers at least 25% of the fracture surface.  (The remaining 75% of the fracture surface may show other angles, such as an acute angle on the same fracture surface as the right angle.) |
|  | Acute angle | Angle between fracture surface and the long axis of the bone is less than 90° and this angle covers at least 25% of the fracture surface. (The remaining 75% of the fracture surface may show other angles, such as an acute angle on the same fracture surface as the right angle.) |
|  | Obtuse angle | Angle between fracture surface and the long axis of the bone is wider than 90° and less than 180°, and this angle covers at least 25% of the fracture surface. (The remaining 75% of the fracture surface may show other angles, such as an acute angle on the same fracture surface as the right angle.) |
| Fracture classification | Post-mortem nTBF | Post-mortem non-traumatic bone fracture, in this study caused by blunt force impact (BFI). |
|  | HIBF | Heat induced bone fracture, fractures caused directly by thermal effects. For example; due to expansion of gasses formed within the bone matrix, or due to shrinkage of the bone structure. |
|  | iHIBF | Indirectly Heat induced bone fracture, fractures that are not directly caused by the heat but would not have formed if the bone did not undergoe heat induced changes. For example; due to combustion bone loses organic components and that results in increased brittleness; any mechanical force applied to the bone after the fire might result in fractures. |

***Table S26 2/2****: Glossary for the used terminology including a description per feature.*


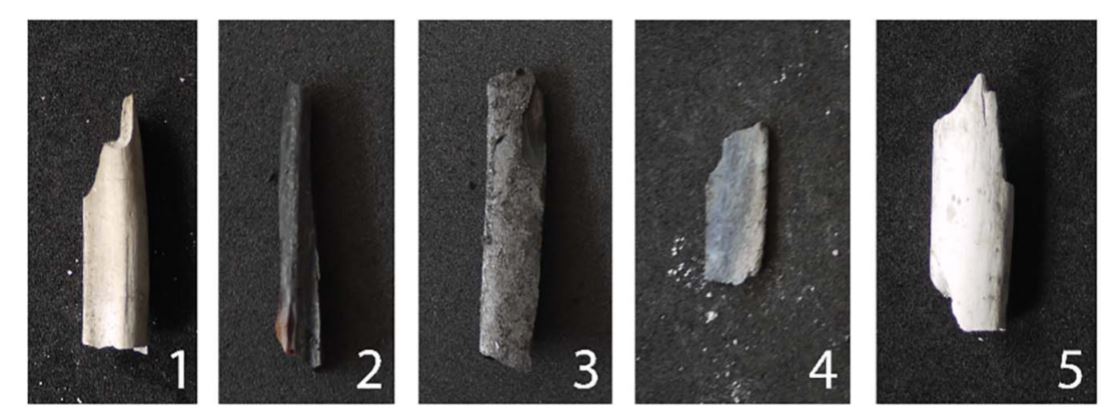


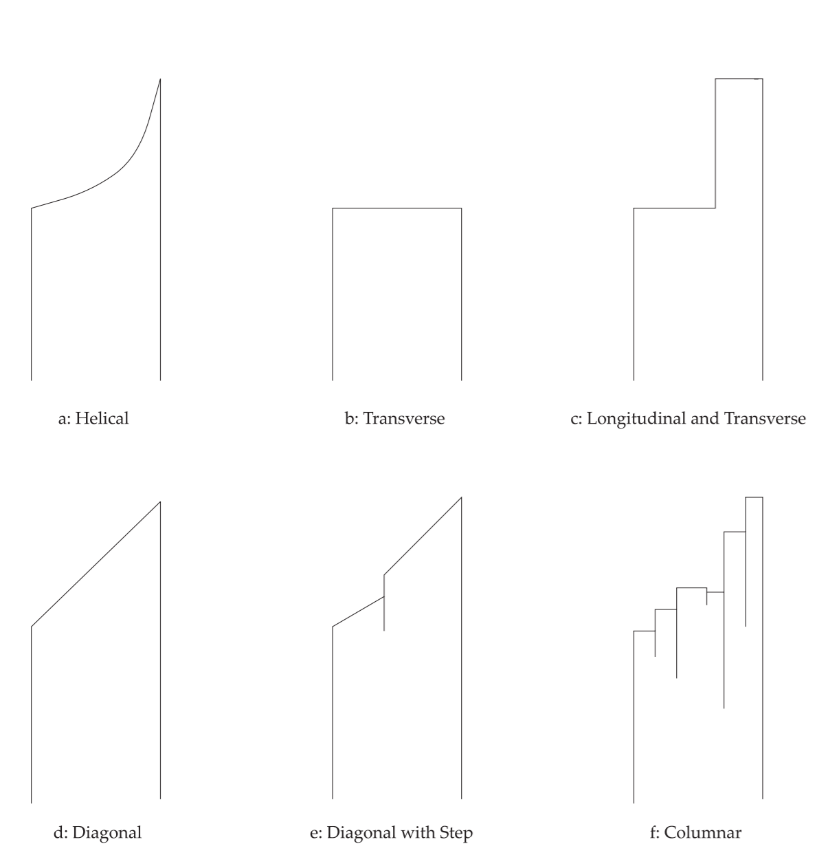
***Fig. S27****: State of burning and Colouration of bone fragments –* ***1****: early carbonization (brown),* ***2****: complete carbonization (black),* ***3****: partially burnt (black to grey/white),* ***4****: partial/early calcination (grey/ grey-blue),* ***5****: complete calcination (white). Image adapted from [36].*


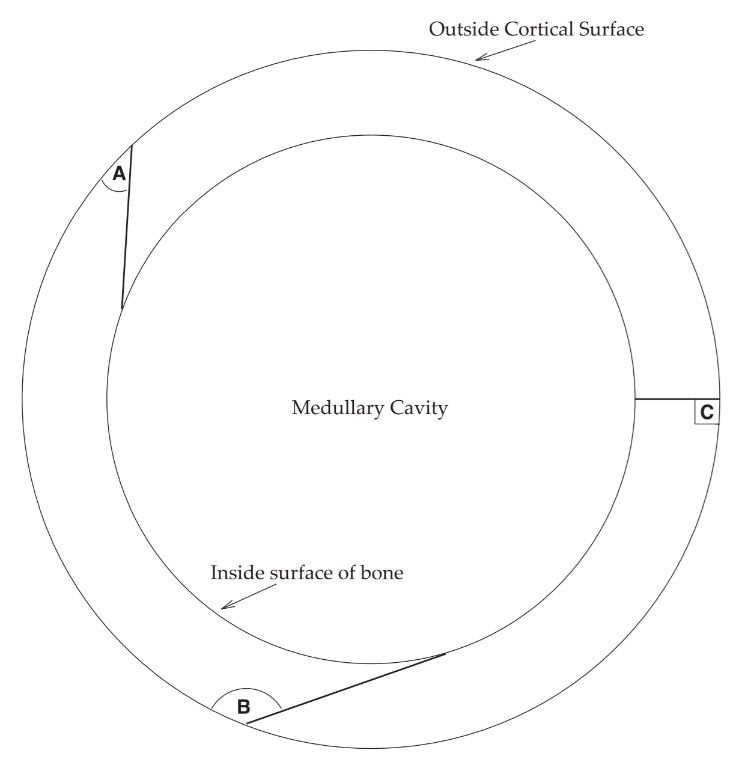


***Fig. S28****:* ***Left:*** *Fracture outline (image adapted from [37]), black arrows indicate examples of shards (extending bone areas from fracture region), red arrow shows sharp edge and yellow arrow shows blunt edge.* ***Right:*** *Fracture angles –* ***A****: acute,* ***B****: obtuse and* ***C****: right angle/perpendicular/transverse (image taken from [37]).*
